# Transcriptome profiling by RNA-Seq reveals differentially expressed genes related to fruit development and ripening characteristics in strawberries (*Fragaria* × *ananassa*)

**DOI:** 10.7717/peerj.4976

**Published:** 2018-06-27

**Authors:** Panpan Hu, Gang Li, Xia Zhao, Fengli Zhao, Liangjie Li, Houcheng Zhou

**Affiliations:** Zhengzhou Fruit Research Institute, Chinese Academy of Agricultural Sciences, Zhengzhou, Henan, China

**Keywords:** Strawberry, Fruit ripening, Transcriptome, Differentially expressed gene

## Abstract

Strawberry (*Fragaria × ananassa*) is an ideal plant for fruit development and ripening research due to the rapid substantial changes in fruit color, aroma, taste, and softening. To gain deeper insights into the genes that play a central regulatory role in strawberry fruit development and ripening characteristics, transcriptome profiling was performed for the large green fruit, white fruit, turning fruit, and red fruit stages of strawberry. A total of 6,608 differentially expressed genes (DEGs) with 2,643 up-regulated and 3,965 down-regulated genes were identified in the fruit development and ripening process. The DEGs related to fruit flavonoid biosynthesis, starch and sucrose biosynthesis, the citrate cycle, and cell-wall modification enzymes played important roles in the fruit development and ripening process. Particularly, some candidate genes related to the ubiquitin mediated proteolysis pathway and MADS-box were confirmed to be involved in fruit development and ripening according to their possible regulatory functions. A total of five *ubiquitin-conjugating enzymes* and 10 *MADS-box transcription factors* were differentially expressed between the four fruit ripening stages. The expression levels of DEGs relating to color, aroma, taste, and softening of fruit were confirmed by quantitative real-time polymerase chain reaction. Our study provides important insights into the complicated regulatory mechanism underlying the fruit ripening characteristics in *Fragaria × ananassa*.

## Introduction

The octoploid strawberry (*Fragaria × ananassa*) is the dominant cultivated specie of its high yield and nutritional value, including vitamin C, sugar and organic acid, and anthocyanin contents (Tanaka, Sasaki & Ohmiya, 2008; Giampieri et al., 2012; Chen et al., 2016b). The strawberry fruit development and ripening process involves intricate metabolic event and is divided into four distinct phases: the green fruit (GF), white fruit (WF), turning fruit (TF), and red fruit (RF) stages (Fait et al., 2008). In the GF stage, fruits undergo cell division and cell expansion. In the WF stage, fruit growth is nearly complete, and fruits begin to enter the maturation process. Subsequently, fruit development enters the TF stage, as indicated by slight coloration. During the RF stage, the characteristics of ripening such as color, aroma, taste, and softening, increase rapidly along with a massive accumulation of pigments, amino acids, and organic acids, among other compounds. In addition, strawberry is an ideal model plant for studying the fruit development and ripening process in non-climacteric fruit (Giovannoni, 2004; Zhang et al., 2011).

Following the sequencing of the genome of diploid woodland strawberry (*Fragaria vesca*) (Shulaev et al., 2011; Edger et al., 2018), the sequence of the octoploid cultivated strawberry (*Fragaria × ananassa*) was also completed (Hirakawa et al., 2014). However, the sequence information of genes published on *Fragaria × ananassa* is insufficient and cannot be wholly used as an available reference genome for studies of the octoploid strawberry in at the molecular level.

The use of transcriptome sequencing technology for gene detection and markers in different strawberry tissues and in response to various environmental stresses has increased (Li et al., 2013; Hollender et al., 2014; Chen et al., 2016a; Wang et al., 2017a). To date, RNA-Seq has also been widely used to study gene expression in the strawberry fruit development and ripening process. The genome-scale transcriptomic analysis of hormone signaling in early strawberry fruit developmental stages from floral anthesis to enlarged fruit suggests that the biosynthesis genes for indole-3-acetic acid (IAA) and gibberellin are most highly and specifically expressed in endosperm and seed coat and play a most prominent role for fruit set (Kang et al., 2013). *FaTCP11*, *FaPCL1-like*, and *FaSCL8* modulate the metabolism of strawberry flavonoids by regulating the expression of flavonoid pathway genes based on a transcriptome correlation network analysis of ripe strawberry fruits (Pillet et al., 2015). Another application of transcriptome analysis in the strawberry anthocyanin biosynthesis pathway reveals that exogenous hematin promotes fruit coloring through multiple related metabolic pathways including anthocyanin biosynthesis and hormone signaling transduction, among others (Li et al., 2016). In red-fruited and natural white-fruited strawberry varieties, transcriptome analysis showed that the genes related to the polyphenol biosynthesis pathway may interact with anthocyanin biosynthesis, flavor formation and fruit softening to regulate the fruit ripening process (Hartl et al., 2017). For postharvest strawberry fruit, transcriptome profiling showed that exogenous IAA delays the fruit ripening process, whereas abscisic acid (ABA) promotes the postharvest ripening by regulating the expressions of genes related to receptor-like kinases, ubiquitin ligases, and IAA and ABA hormone signaling pathways (Chen et al., 2016b). Transcriptomic analysis of strawberry endogenous IAA suggests that the candidate genes of *FaTAA1*, *FaTAR2*, *FaAux/IAA11*, and *FaARF6a* are involved in active IAA biosynthesis in the strawberry ripe receptacle (Estrada-Johnson et al., 2017). RNA-Seq is also used to study the polymorphisms of the octoploid strawberry. According to transcriptional analyses of the *FaERF* family in ripening strawberry fruits, *FaERF3*, *FaERF6*, and *FaERF71a* as candidates were identified to play a primary role in the ripening receptacle (Sanchez-Sevilla et al., 2017). A recent study suggests that the down regulation of the key gene *PDHE1α* of the pyruvate dehydrogenase for glycolysis derived oxidative phosphorylation inhibits respiration and ATP biosynthesis but promotes the accumulation of sugar, ABA, ethylene (ETH) and polyamines, and ultimately accelerates the strawberry fruit ripening (Wang et al., 2017b).

The functions of the ubiquitin mediated proteolysis pathway in the regulation of fruit ripening have been studied in banana, tomato, papaya, and barbarum, and these studies confirm the regulatory role of the ubiquitin proteasome in the fruit ripening process (Liu et al., 2013; Wang et al., 2014; Bi et al., 2015; Zeng et al., 2015). Research on the regulatory mechanism of fruit ripening indicates that MADS-box transcription factors have a pivotal effect on fruit ripening by regulating carotenoid synthesis, the ETH signaling pathway, cell wall metabolism, flavonoid and lignin biosynthesis, and cuticle development in apple, banana, tomato, and peach (Youssef et al., 2012; Ireland et al., 2013; Liu et al., 2013; Feng et al., 2016; Garceau, Batson & Pan, 2017; Yin et al., 2017; Hu et al., 2017).

In this study, based on the characteristics changes in fruit development and ripening, a global expression analysis by RNA-Seq at four stages of strawberry fruit ripening was performed to discover additional candidate genes in ubiquitin mediated proteolysis for MADS-box transcription factors and for other aspects. In this paper, the different expression patterns of differentially expressed genes (DEGs) related to coloring, aroma, taste, softening, and other aspects among different fruit ripening stages in strawberry are outlined. The purpose of this study was to understand the molecular mechanisms controlling the characteristics of strawberry fruit ripening according to transcriptome profiling analysis and to provide a theoretical foundation for the cultivation of strawberry varieties.

## Materials and Methods

### Plant materials

**Figure 1:**
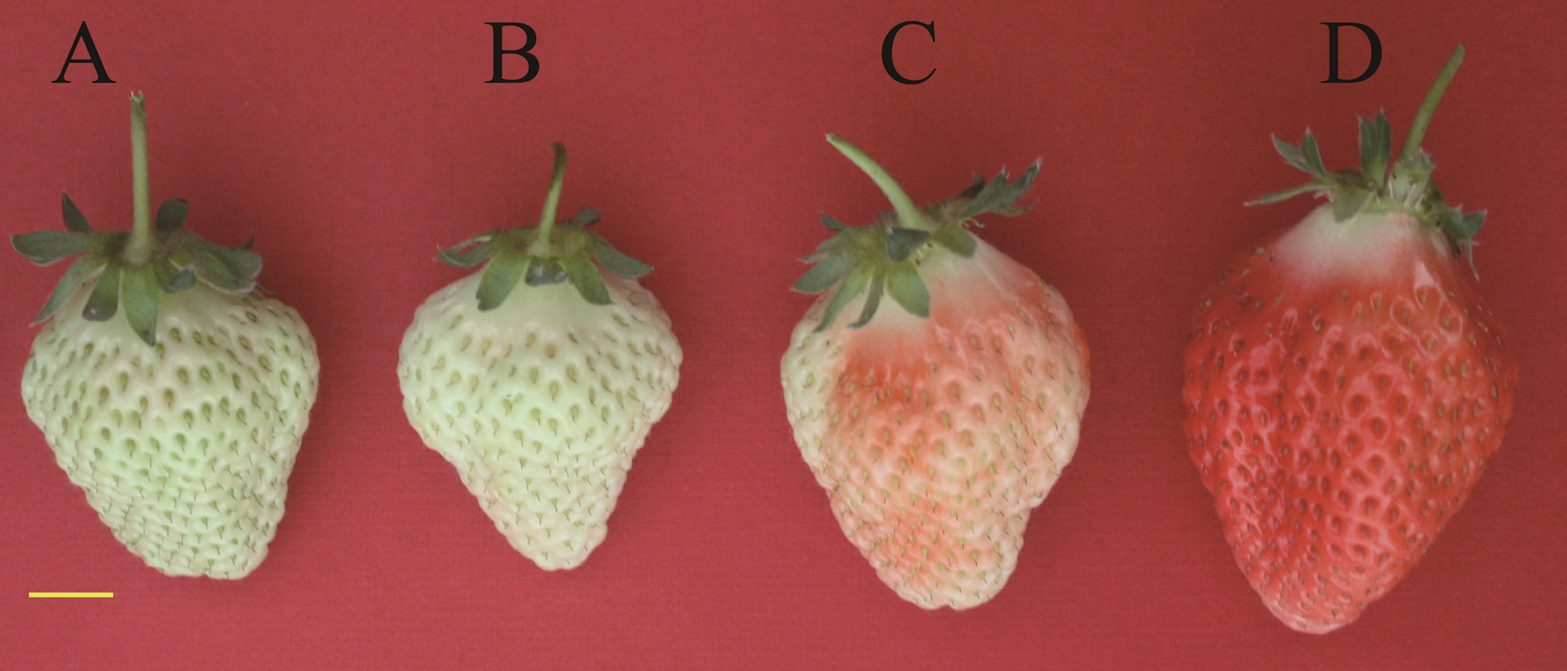
Tissues of strawberry “Toyonoka” used in deep sequencing. (A) l-GF; (B) WF; (C) TF; (D) RF. Yellow bar = 10 mm. Photo credit: Panpan Hu.

The fruits used in this study were obtained from the strawberry cultivar “Toyonoka” cultivated in the greenhouse (8–28 °C, relative humidity 55–70%, and without supplemental lighting) in Zhengzhou, Henan, China. Fruits of large green fruit (l-GF), WF, TF, and RF stages were selected as the sequencing materials (Fig. 1). A total of 10 uniformly sized fruits were sampled at each stage. For quantitative real-time polymerase chain reaction **(**qRT-PCR), the GF stage was subdivided into small green fruit (s-GF), middle green fruit (m-GF), and l-GF stages. In total, fruits of six different ripening stages (s-GF, m-GF, l-GF, WF, TF, and RF) were prepared for qRT-PCR. Three uniform fruits were sampled at each of the six stages for RNA isolation and cDNA synthesis (three replicates). The experimental materials were placed immediately in liquid N_2_ and stored at −80 °C for RNA extraction.

### Total RNA extraction, library preparation, and transcriptome sequencing

Total RNA was extracted using a Spin Column Plant total RNA Purification Kit (Order No. B518661; Sangon Biotech, Shanghai, China) according to the manufacturer’s instructions. DNase digestion with Dnase I (Promega, Madison, WI, USA) was performed to remove contaminating DNA. Briefly, mRNA was purified from total RNA using poly-T oligo-attached magnetic beads (Novogene, Beijing, China) and then broken into short fragments. With these fragments as templates, cDNA were synthesized. To select cDNA fragments of 150–200 bp in length, the library fragments were purified with an AMPure XP system (Beckman Coulter, Beverly, MA, USA). Then, those fragments were selected for PCR amplification as sequencing templates. The PCR products were purified and library quality was assessed on an Agilent Bioanalyzer 2100 system (Agilent Technologies, Santa Clara, CA, USA). The clustering of the index-coded samples was performed on a cBot Cluster Generation System using a TruSeq PE Cluster Kit v3-cBot-HS (Illumina, Santiago, CA, USA) according to the manufacturer’s instructions. After cluster generation, the library preparations were sequenced on an Illumina HiSeq 4000 platform (Novogene, Beijing, China) and paired-end reads were generated. Each RNA sample was ligated with a separate adapter and sequenced together in a single run.

### Data processing, transcriptome assembly, and functional annotation

The raw image data files from the Illumina HiSeq 4000 were transformed into the original sequenced reads (raw reads) by CASAVA 1.8 base calling analysis and processed through in-house Perl scripts. Clean data (clean reads) were obtained by eliminating the low-quality reads (reads containing an adapter, reads containing poly-N, and reads with Qphred ≤ 20, equivalent to reads with base call accuracy less than 99%) from raw reads. Transcriptome assembly was accomplished based on the transcripts and unigenes using Trinity (Grabherr et al., 2011) with min_kmer_cov set to 2 by default and all other default parameters set. The clean data with high quality was spliced to get the reference sequence (transcript) for subsequent analysis.

Gene functions were annotated based on the following seven databases (Table S1): NCBI non-redundant protein sequences (Nr), NCBI non-redundant nucleotide sequences (Nt), Protein family, EuKaryotic Orthologous Groups (KOG), a manually annotated and reviewed section of the UniProt Knowledgebase database (Swiss-Prot), KEGG Ortholog, and Gene ontology (GO). The URLs, annotation methods and parameters of the seven databases are shown in Table S1, and the information of all software versions and parameters is shown in Table S2.

### Differentially expression analysis

Clean reads of each library were compared with transcriptome reference sequences. Gene expression levels were evaluated by RNA-Seq by Expectation Maximization with the bowtie2 parameters (Li & Dewey, 2011) for each sample (Table S2). The read_count for each gene was obtained from the mapping results of clean reads back onto the assembled transcriptome. The read_count of each gene was normalized data of the fragments per kilobase of exon per million fragments mapped (FPKM) which is the most commonly used method of estimating gene expression levels (Trapnell et al., 2010). Those genes whose FPKM > 0.3 were considered to be expressed (Fig. S1; Table S3) (Mortazavi et al., 2008; Trapnell et al., 2010; Ho et al., 2012). For those samples with biological replicates, differential expression of unigenes was analyzed and calculated based on the read_count value using the DESeq R package (Anders & Huber, 2010). Based on the negative binomial distribution model, DESeq provided statistical routines for determining differential expression in digital gene expression data. The *p*-values in statistics were adjusted using Benjamini and Hochberg’s approach for controlling the false discovery rate (Benjamini & Hochberg, 1995). The thresholds for judging significant difference of gene expression level between any two groups were padj < 0.05 and log_2_ (fold change) ≥ 1 or log_2_ (fold change) ≤ −1. The *p*-adjusted (padj) was the corrected *p*-value, and a small padj value of DEG indicated high significance of the differential expression.

### qRT-PCR analysis

Total RNA extraction and reverse transcription PCR were performed as previously described for RNA extraction and library preparation of RNA-Seq. All qRT-PCR samples were run on a Light Cycler 480 system (Roche, Basel, Switzerland). Each reaction was performed with a total volume of 20 μL that contained 5 μL of first-strand cDNA as a template, with a pre-incubation program of 5 min at 95 °C, followed by 45 cycles of 10 s at 95 °C and 30 s at 60 °C, according to the Light Cycler 480 SYBR Green I Master protocol (Cat. No.04707516001). Gene-specific primers were designed with Primer Premier 5 (Table S4). The *FaACTIN* gene was used as an internal reference for gene expression. Gene expression levels were calculated using the 2^−ΔΔCt^ method (Livak & Schmittgen, 2001). The mean threshold cycle values for each gene were obtained from three independent PCR reactions.

## Results

### RNA-Seq

A total of 45.48 G of data with two biological replicates of each library was generated in this study (Table 1). A total of 172,799 transcripts including isoforms assembled by Trinity (Grabherr et al., 2011) were obtained based on the raw reads with an average length of 951 bp. Then, 91,790 valid unigenes were obtained (Supplementary File S1), with an average length of 714 bp. Figure S2 shows the length distributions of the transcripts and unigenes.

**Table 1:** Throughput and quality of RNA-Seq data.

Sample	Raw reads	Clean reads	Clean bases	Error rate (%)	*Q*20^a^ (%)	*Q*30^b^ (%)	GC (%)
l-GF1	58541836	57209502	8.58G	0.02	96.48	91.19	46.74
l-GF2	60581866	59222064	8.88G	0.02	96.27	90.72	46.93
WF1	66696962	65070548	9.76G	0.02	96.56	91.35	46.8
WF2	61783100	60066380	9.01G	0.02	96.55	91.29	46.84
TF1	63081374	61671990	9.25G	0.02	96.55	91.29	46.66
TF2	61345068	59832880	8.97G	0.02	96.48	91.16	46.42
RF1	61847198	60455548	9.07G	0.02	96.68	91.53	46.04
RF2	59579024	58261550	8.74G	0.02	96.67	91.53	45.88

**Note:**

a,b Q20 and Q30 indicate the percentage of bases whose Qphred > 20, 30. Error rate, Q20, Q30 and GC content distribution are used to reflect the quality of sequencing data.

### Functional annotation of unigenes

Of the total 91,790 unigenes, 57,200 unigenes were annotated to the seven databases (Table S5). Among all the databases, 40.53% of unigenes were aligned to the Nr protein database with an *e*-value threshold of *e^−^*^5^. The similarity of gene sequence and the function information of genes between strawberry and other species were obtained through the Nr annotation database. The results of species classification, *e*-value distribution, and sequence similarity distribution are shown in Figs. S3A–S3C, respectively.

Gene ontology annotation results primarily describe gene functions. A total of 26,523 unigenes in the GO database were classified into 57 functional categories, among which 22,087 unigenes were assigned to biochemical processes, 10,259 genes were assigned to cellular components, and 16,418 unigenes were assigned to molecular functions (Table S6).

To evaluate the effectiveness of the annotation process and possible functions of unigenes, 13,442 unique sequences were annotated to the KOG database, based on their ortholog relationship. KOG was segmented into 26 orthologous groups (Table S7). Among the 26 KOG groups, 2,263 and 1,808 unigenes were enriched to the “general function prediction only” and “post-translational modification, protein turnover, chaperones” clusters, respectively. Based on the same ortholog gene function in the KOG classification, we could effectively analyze the functions of DEGs in fruit ripening.

The KEGG database is available to systematically analyze the metabolic pathways and functions of gene products and compounds in cells by integrating the genome, molecular chemical and biochemical systems data. Annotated to the KEGG database, 10,932 unigenes were assigned to 274 KEGG pathways using BLASTx with an *e*-value threshold of *e^−^*^10^ (Table S8). KEGG results provided a good transcription platform for investigating the related metabolic pathways in the strawberry development and ripening process.

### Analysis of differentially expressed genes in the fruit development and ripening process

**Figure 2:**
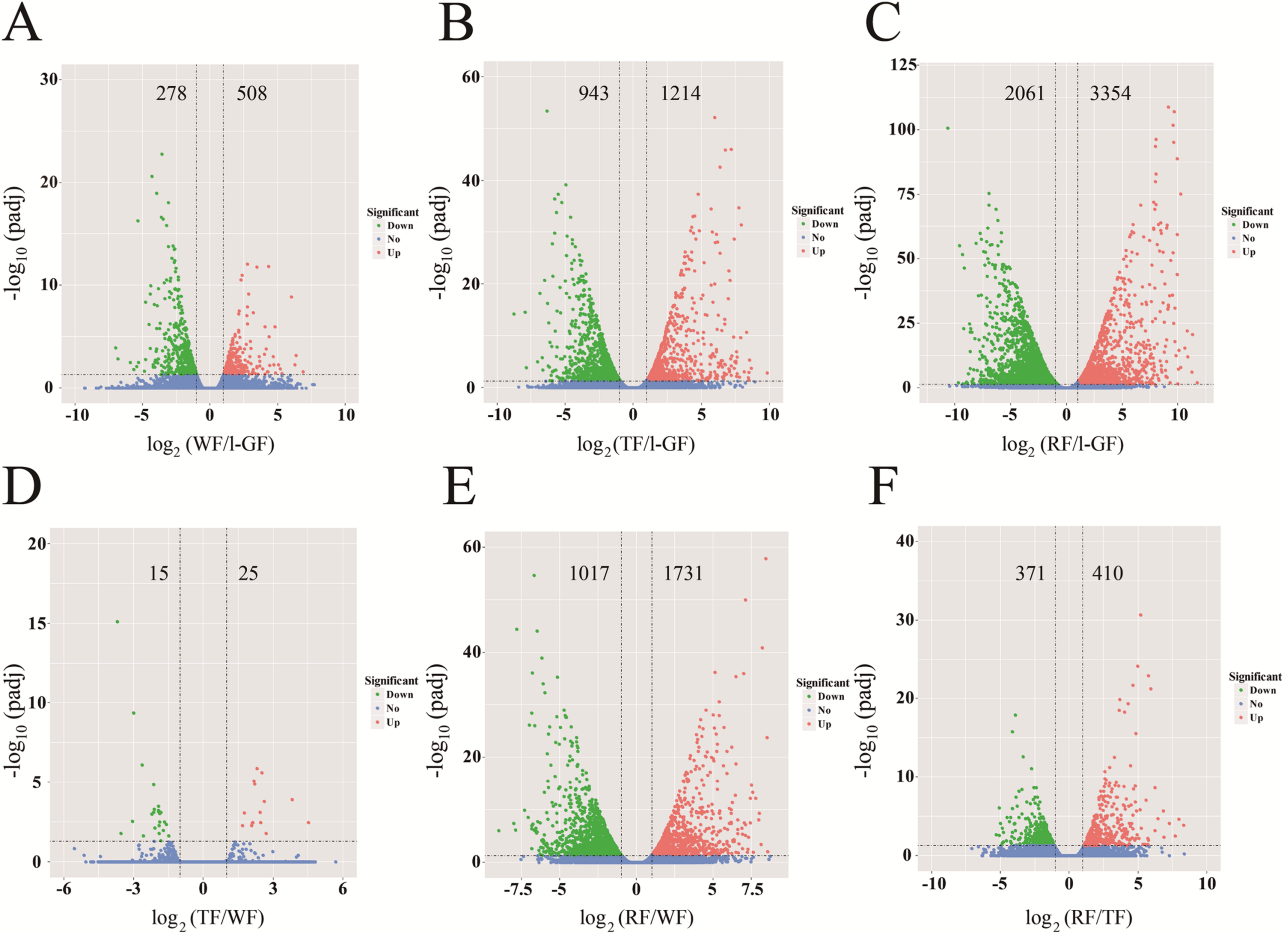
The volcano plots of DEGs in six combinations. (A) WF/l-GF; (B) TF/l-GF; (C) RF/l-GF; (D) TF/WF; (E) RF/WF; (F) RF/TF. The *x-axis* represents the gene expression times. The *y-axis* represents the statistically significant degree of gene expression change. The smaller the corrected *p*-value, the larger the −log_10_ (padj), and the more significant the difference. The scattered dots represent each gene, the blue dots indicate genes with no significant differences, the red dots indicate up-regulated genes with significant differences, and the green dots indicate down-regulated genes with significant differences.

**Figure 3:**
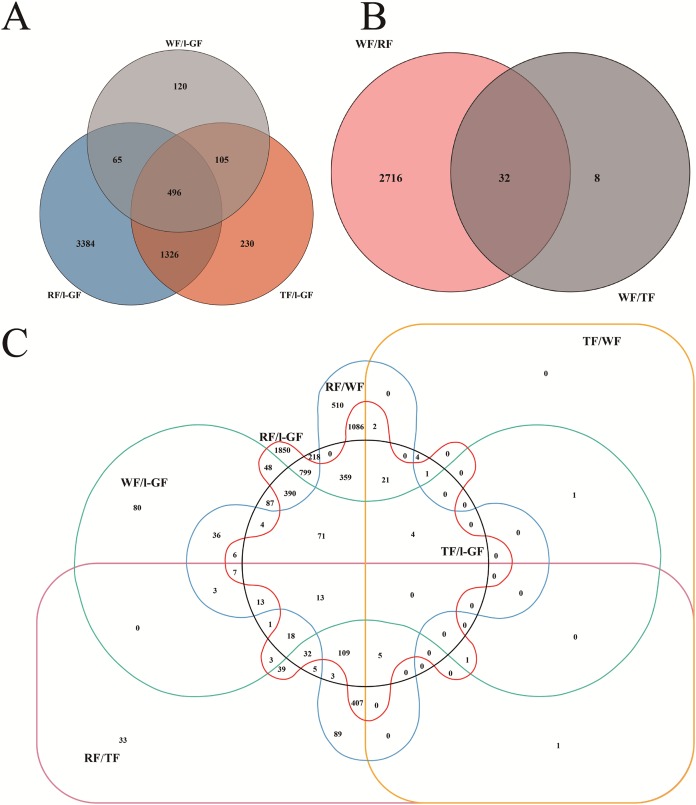
Venn diagrams for the different DEGs between each combination. (A) Number of common and specific DEGs in WF/l-GF, TF/l-GF, and RF/l-GF. (B) Number of common and specific DEGs in TF/WF and RF/WF. (C) Number of common and specific DEGs in six combinations (WF/l-GF, TF/l-GF, RF/l-GF, TF/WF, RF/WF, and RF/TF). The sum of the numbers in each large circle represents the total number of DEGs in the comparison, and the overlapping parts of the circle represent the number of common DEGs among the combinations.

In different comparative combinations, volcano plot (Figs. 2A–2F; Table S9) can visually demonstrate the relationship among padj, log_2_ (fold change) and the number of up/down-regulated DEGs. A total of 6,608 DEGs with 2,643 up-regulated and 3,965 down-regulated, were differentially expressed in the six combinations (WF/l-GF, TF/l-GF, RF/l-GF, TF/WF, RF/WF, and RF/TF). The number of up/down-regulated DEGs in each combination is displayed in Figs. 2A–2F, which shows that the most DEGs were detected when comparing l-GF with RF and TF and WF with RF. The WF and TF libraries possessed similar gene expression patterns, and therefore the fewest DEGs were detected in the TF/WF combination. Of these DEGs, in each combination, the genes were predominantly down-regulated. For the different combinations, Fig. 3A–3C shows the numbers of specific and common DEGs. In the comparison of l-GF with WF, TF, and RF, 785, 2,157, and 5,271 DEGs were identified, respectively (Fig. 3A). In the comparison of WF with TF and RF, 40 and 2,748 DEGs were identified (Fig. 3B). Compared TF with RF, 781 DEGs were identified (Fig. 3C). Subsequent analyses focused on these DEGs related to fruit development and ripening characteristics.

### Enrichment pathway analysis of DEGs

The functional enrichment analyses of DEGs are based on the GO and KEGG databases. GO enrichment analysis of the DEGs was performed by the GOseq R packages (Young et al., 2010). KEGG (Kanehisa et al., 2008) enrichment analysis was used to test the statistical enrichment of DEGs with KOBAS software (Mao et al., 2005). GO and KEGG pathway enrichment analyses (padj < 0.05) were used to categorize the biological functions of DEGs. The expression patterns of the DEGs and their enrichment results in different combinations showed that the down-regulated expression of DEGs and metabolic pathways was predominant in the strawberry fruit development and ripening process.

### Genes related to color, aroma, taste, and softening in the fruit development and ripening process

**Figure 4:**
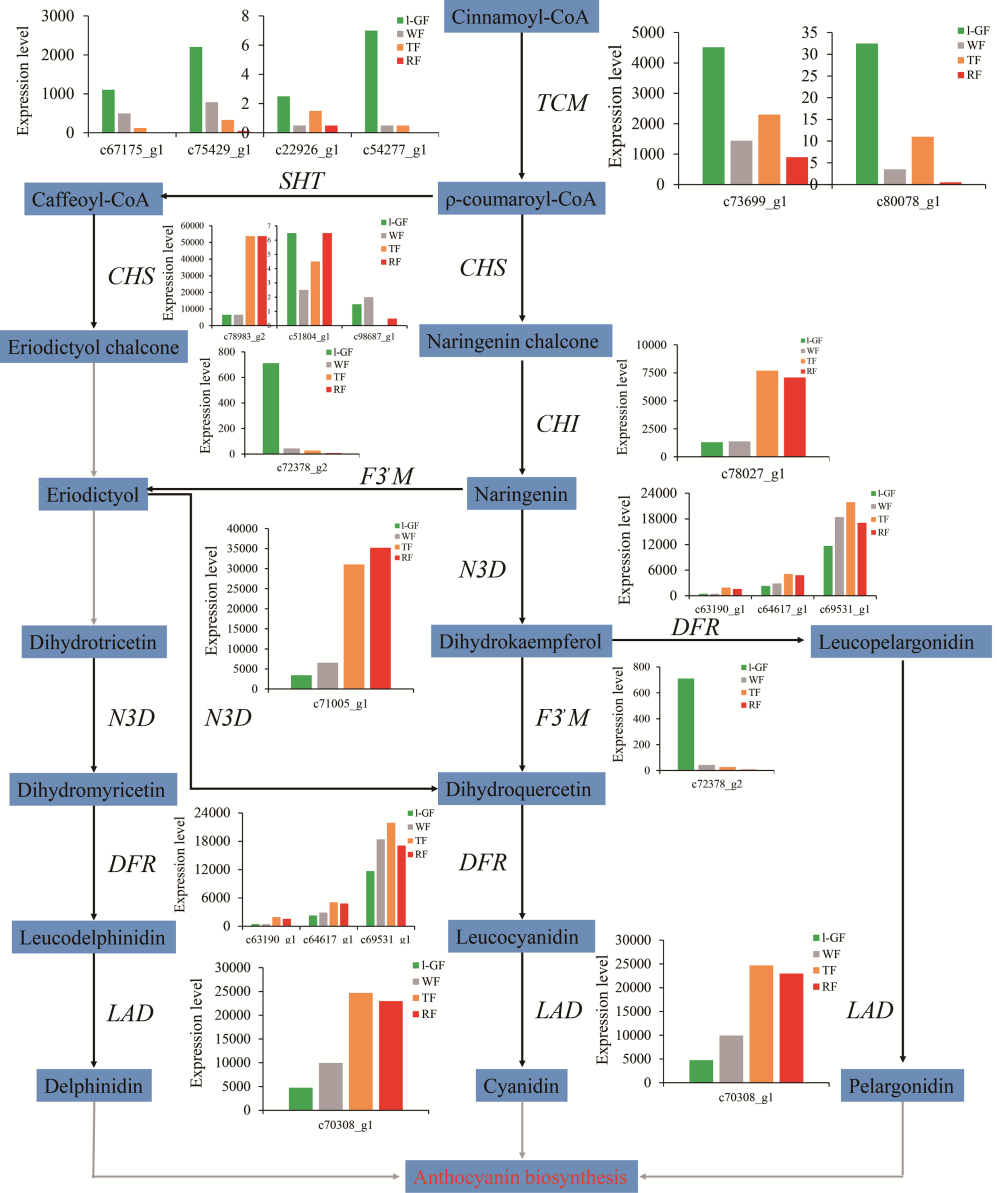
The expression pattern of genes involved in anthocyanin biosynthesis. Enzyme names, unigene ids and expression patterns are indicated on each step. The *y-axis* represents average read_count value of each library. No gene is found in the grey line step. Study sites: TCM, trans-cinnamate 4-monooxygenase; SHT, shikimate O-hydroxycinnamoyl transferase; CHS, chalcone synthase; CHI, chalcone isomerase; F3*′*M, flavonoid 3*′*-monooxygenase; N3D, naringenin 3-dioxygenase; DFR, bifunctional dihydroflavonol-4-reductase; LAD, leucoanthocyanidin dioxygenase; ANS, anthocyanidin reductase.

Research into non-climacteric fruit color is concentrated on flavonoid biosynthesis, and the types of anthocyanins in strawberry are pelargonidin, delphinidin, and cyanidin (Fig. 4). In this study, the expression of most genes in anthocyanin biosynthesis such as *chalcone synthase* (*CHS*) (c51804_g1, c78983_g2, and c98687_g1), *chalcone isomerase* (*CHI*) (c78027_g1), *naringenin 3-dioxygenase* (c71005_g1), *dihydroflavonol-4-reductase (DFR*) (c63190_g1, c64617_g1, and c69531_g1), and *leucoanthocyanidin dioxygenase* (c70308_g1) were up-regulated with strawberry ripening (Fig. 4). However the down-regulated expression of *flavonoid 3′-monooxygenase* (*F3′M*) (c72378_g2) decreased the synthesis of cyanidin and accelerated the accumulation of pelargonidin in anthocyanin biosynthesis (Fig. 4). Cluster analysis was used to analyze 36 unigenes involved in flavonoid biosynthesis using the expression data (read_count) provided in Table S10 (Fig. S4A). Among the 36 genes, the relative expression analysis, which revealed the expression patterns of genes over time, showed that the expression of two gene was up-regulated and that of six genes was down-regulated during fruit ripening (Fig. S4B; Table S11). Figure S4C showed the differential expression patterns of 10 DEGs in flavonoid biosynthesis (Table S11), and most DEGs played important roles in anthocyanin biosynthesis (Fig. 4).

The MYB-bHLH-WD40 transcription complex also regulates anthocyanin biosynthesis. Of the genes encoding MYB transcription factors in this data set, one up-regulated unigene was *R2R3 MYB transcription factor* (*FaMYB10*) (c76851_g2), which can positively control the biosynthesis of anthocyanin (Lin-Wang et al., 2014; Medina-Puche et al., 2014). Among the bHLH transcription factors, two down-regulated unigenes (c75633_g2 and c78773_g1) were annotated as bHLH33 and bHLH3, respectively, which can interact with MYB10 to play important roles in proanthocyanidin and anthocyanin biosynthesis (Schaart et al., 2013). Figures S5A and S5C shows the up-regulated and down-regulated expression of MYB and bHLH transcription factor genes (Table S11), and between of different combinations, 16 and 21 DEGs were found (Figs. S5B and S5D; Table S11). The two DEGs MYB113-like (c76114_g1) and R2R3 MYB transcription factor (c76851_g2) were up-regulated with strawberry ripening. The down-regulated DEGs of MYB transcription factors included MYB39-like, MYB12-like, and MYB1R1-like (c67743_g1, c68086_g1, and c75011_g1). A total of three DEGs of transcription factors bHLH104-like, bHLH135-like, and bHLH122-like (c71077_g1, c76460_g2, and c78358_g1, respectively) were up-regulated, although more DEGs were down-regulated including bHLH33 (c75633_g2). The expression pattern of these genes was closely related to fruit coloring. A total of 10 WD40 repeat-containing protein genes were described in the RNA-Seq data, but none had significantly differential expression at the four fruit ripening stages.

**Figure 5:**
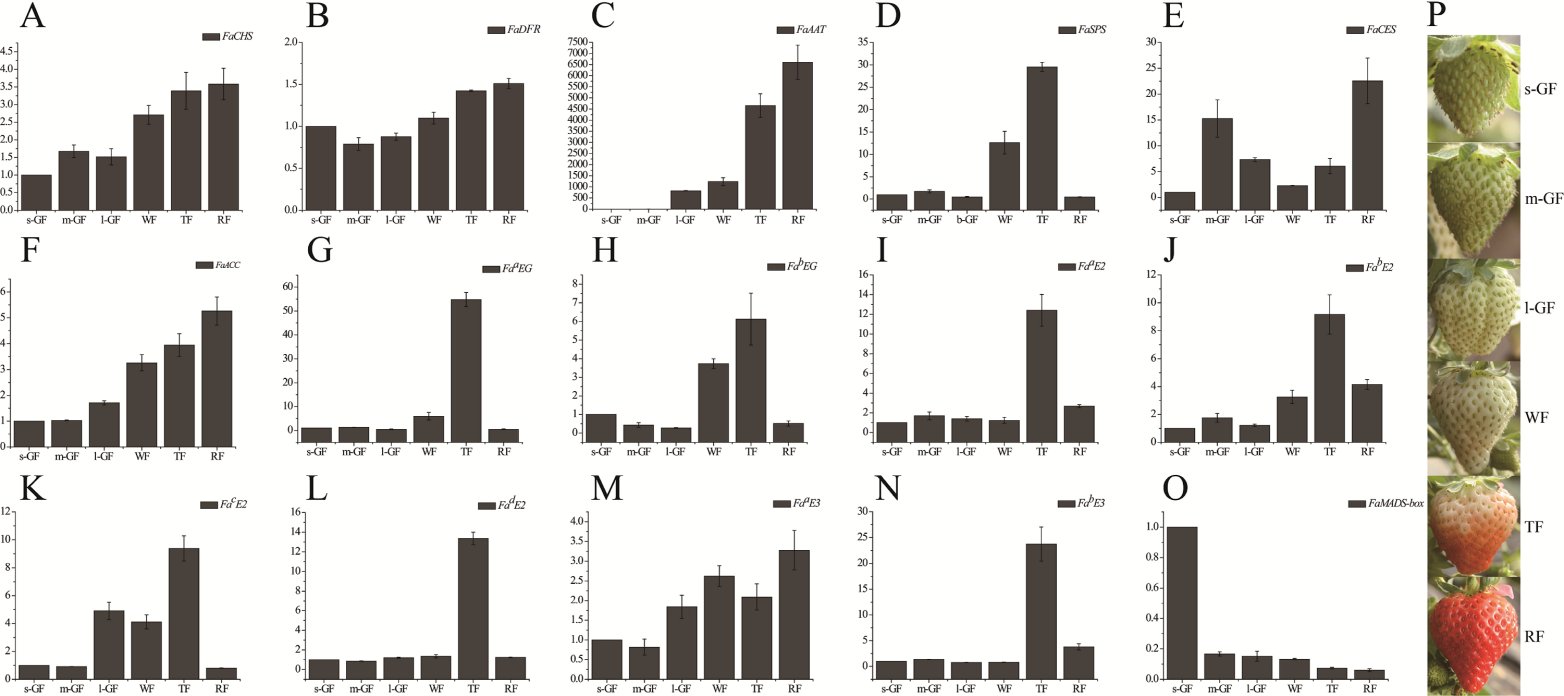
Expression profile of candidate genes during different fruit development and ripening stages in qRT-PCR. qRT-PCR analysis of strawberry candidate gene (A) *FaCHS;* (B) *FaDFR;* (C) *FaAAT;* (D) *FaSPS*; (E) *FaCES*; (F) *FaACC*; (G) *Fa^a^EG*; (H) *Fa^b^EG*; (I) *Fa^a^E2*; (J) *Fa^b^E2*; (K) *Fa ^c^E2*; (L) *Fa ^d^E2*; (M) *Fa ^a^E3*; (N) *Fa ^b^E3*; (O) *FaMADS-box*. (P) Tissues of strawberry ‘Toyonoka’ used in qRT-PCR. CHS, chalcone synthase; DFR, bifunctional dihydroflavonol 4-reductase; AAT, alcohol acyltransferase; SPS, sucrose-phosphate synthase 1; CES, cellulose synthase A catalytic sub-unit 4; ACC, Acetyl-coenzyme A carboxylase carboxyl transferase sub-unit alpha; ^a^EG, endoglucanase CX-like; ^b^EG, endoglucanase 24-like; ^a^E2, ubiquitin-conjugating enzyme E2 5-like; ^b^E2, ubiquitin-conjugating enzyme E2 23-like; ^c^E2, ubiquitin-conjugating enzyme E2 28-like; ^d^E2, ubiquitin-conjugating enzyme E2 4-like; ^a^E3, E3 ubiquitin-protein ligase UPL3-like; ^b^E3, cullin-1-like; MADS-box, MADS-box protein ZMM17-like. *FaActin* were used as an internal control. Result shows expression value of candidate genes relative to s-GF stage. The experiments were repeated three times and provided consistent results. The mean values and error bars were obtained from three biological and three technical replicates.

Differentially expressed genes of *CHS* (c78983_g2) and *DFR* (c63190_g1) involved in anthocyanin synthesis were verified by qRT-PCR (Figs. 5A and 5B). The results showed that the expression levels of genes were consistent with the results of transcriptome analysis (Figs. S6A and S6B): their expression levels increased and promoted the biosynthesis and accumulation of anthocyanin during fruit ripening.

**Figure 6:**
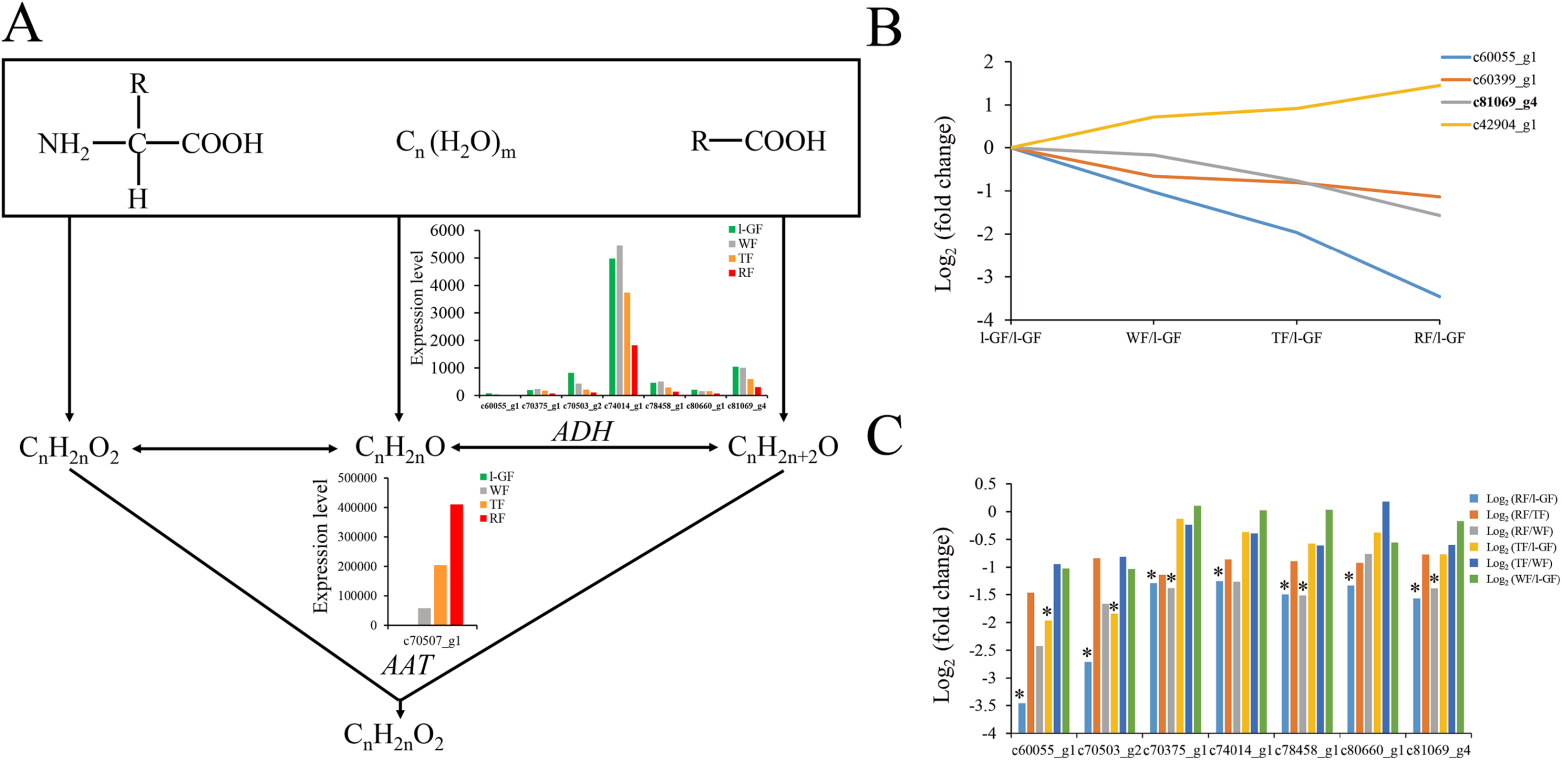
The expression pattern of genes involved in ester biosynthesis. (A) Ester biosynthesis pathway. Enzyme names, unigene ids and expression patterns are indicated on each step. The *y-axis* represents average read_count value of each library. ADH, alcohol dehydrogenase; AAT, alcohol acyltransferase. (B) The relative expression of down-regulated genes in the degradation of aromatic compound pathway. Black fonts indicate the up-regulated gene ID. (C) The expression pattern of DEGs in the degradation of aromatic compound pathway. The asterisk (*) indicates that the gene is satisfied the differentially expression analysis criteria (padj < 0.05 and log_2_ (fold change) ≥ 1 or log_2_ (fold change) ≤ −1) in the corresponding comparative combination.

Strawberry fruit is rich in characteristic aromatics in the later stages of fruit ripening. The primary aromatic compounds are derived from ester metabolism. The precursors of esters such as amino acids, sugars and lipids are converted to acids, alcohols, and aldehydes in ester biosynthesis (Fig. 6A). The decreased expression level of *alcohol dehydrogenase* (*FaADH*) (c60055_g1, c70375_g1, c70503_g2, c74014_g1, c78458_g1, c80660_g1, and c81069_g4) with strawberry fruit ripening (Fig. 6A) is consistent with previous studies on peach *PpADH1*, *PpADH2*, and *PpADH3* (Zhang et al., 2010). The expression level of *alcohol acyltransferase* (*FaAAT*) (c70507_g1) was significantly difference in the fruit ripening process, and the expression values in WF, TF, and RF increased to 49.5, 174.5, and 380.8 times, respectively, than those in l-GF (Fig. S6C; Table S11). The qRT-PCR result for *FaAAT* showed a significant increase during fruit ripening (Fig. 5C); therefore, the *FaAAT* gene was considered to play a vital role in the metabolism of esters. To study the functions of additional genes on aromatics, the expression patterns of genes in the degradation of aromatic compound pathways was analyzed based on the transcriptome data (Figs. 6B and 6C; Table S11), and all those genes were down-regulated during fruit ripening.

**Figure 7:**
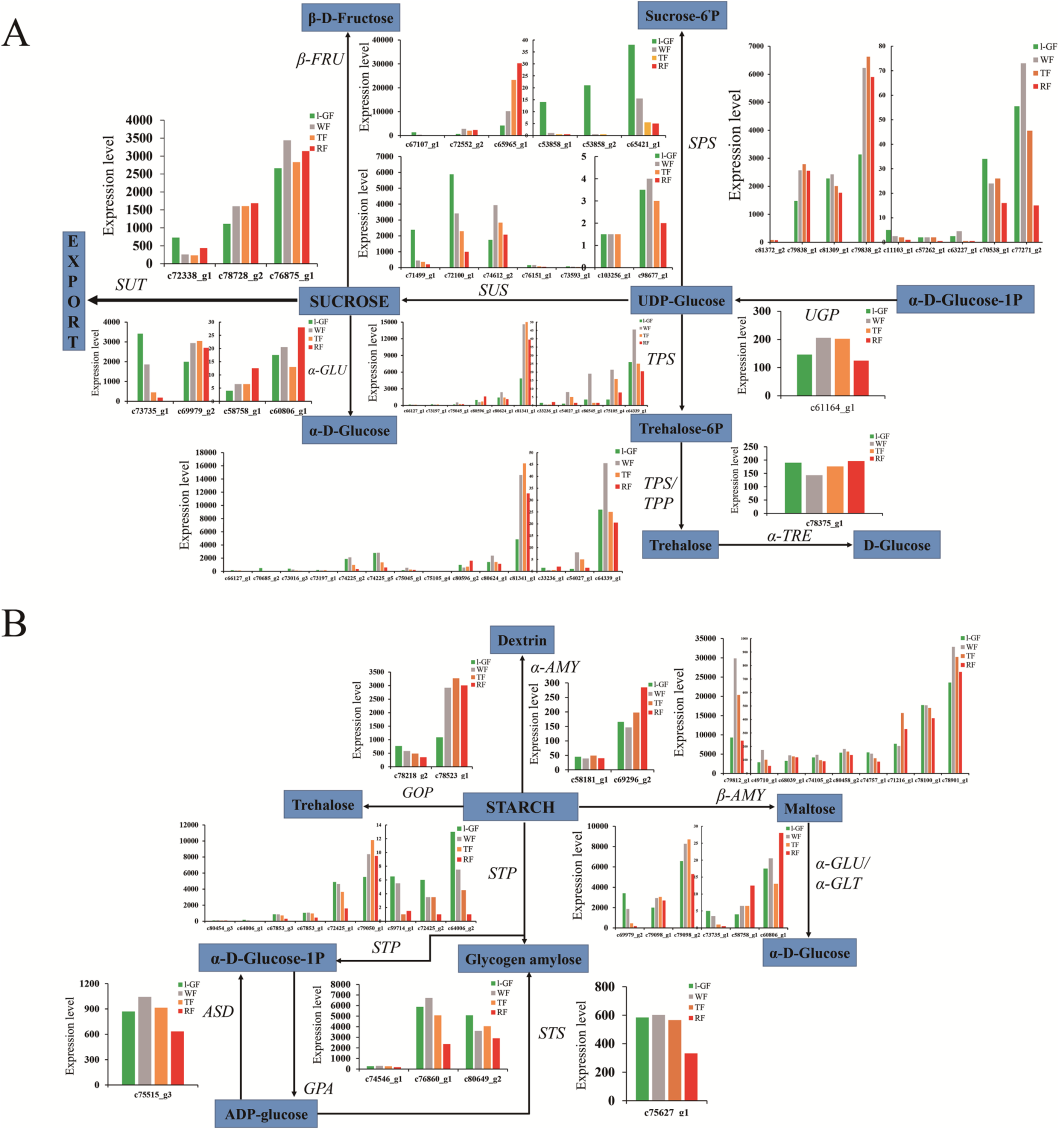
The expression pattern of genes involved in starch and sucrose biosynthesis. (A) Sucrose biosynthesis pathway. (B) Starch biosynthesis pathway. Enzyme names, unigene ids and expression patterns are indicated on each step. The *y-axis* represents average read_count value of each library. Study sites: β-FRU, β-fructofuranosidase; α-GLU, α-glucosidase; SUT, sucrose translocase; SUS, sucrose synthase; SPS, sucrose-phosphate synthase; TPS, trehalose 6-phosphate synthase; TPP, trehalose 6-phosphate phosphatase; α-TRE, α-trehalase; UGP, UTP—glucose-1-phosphate uridylyltransferase; ASD, ADP-sugar diphosphatase; STP, starch phosphorylase; GPA, glucose-1-phosphate adenylyltransferase; STS, starch synthase; GOP, glycogen operon protein; α-AMY, α-amylase; β-AMY, β-amylase; α-GLU, α-glucosidase.

**Figure 8:**
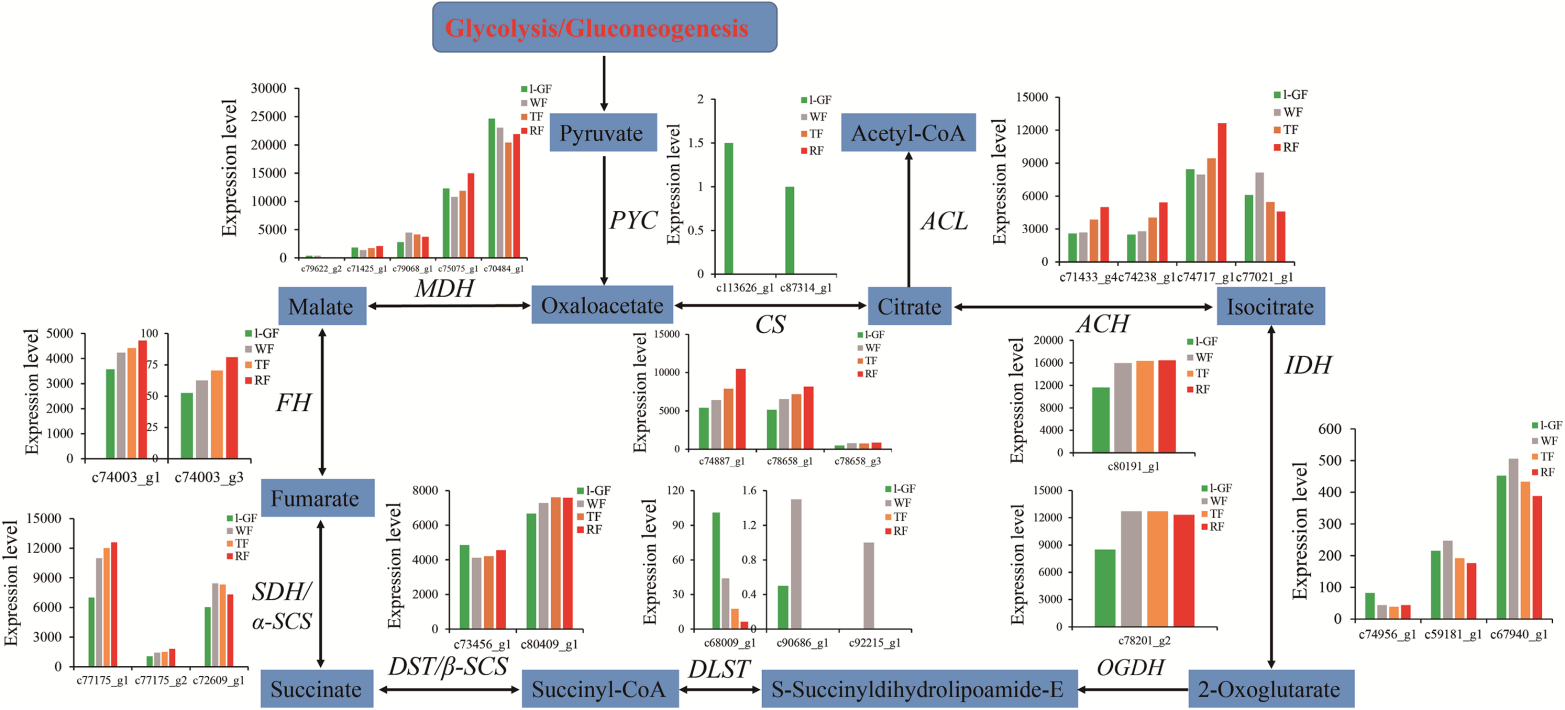
The expression pattern of genes involved in citrate cycle. Enzyme names, unigene ids and expression patterns are indicated on each step. The *y-axis* represents average read_count value of each library. Study sites: PYC, acetyl-CoA C-acetyltransferase; MDH, malate dehydrogenase; FH, fumarate hydratase; SDH/α-SCS, succinate dehydrogenase/succinyl-CoA synthetase alpha subunit; DST/β-SCS, dihydrolipoamide succinyltransferase/succinyl-CoA synthetase beta subunit; DLST, dihydrolipoamide succinyltransferase; OGDH, 2-oxoglutarate dehydrogenase E1 component; IDH, isocitrate dehydrogenase; ACH, aconitate hydratase; CS, citrate synthase; ACL, ATP citrate (pro-S)-lyase.

Sugar and acidity are the primary components of fruit soluble solids governing fruit quality, which depend on starch and sucrose metabolism (Fig. 7) and citrate cycle metabolic pathways (Fig. 8), respectively. In the qRT-PCR, the up-regulated *SPS 1-like* (*FaSPS*) (c79838_g1) had the highest level in RF (Fig. 5D). The down-regulated *FaCES* (c75759_g1) decreased from l-GF to WF but increased from WF to RF (Fig. 5E). The up-regulated expression of *FaACC* (c77811_g1) in the qRT-PCR was consistent with the transcriptome expression pattern (Fig. 5F). The expression patterns of *FaSPS*, *FaCES*, and *FaACC* in transcriptome data were shown in Fig. S6D–S6F. Confirming that the expression levels of most genes decreased during fruit ripening, Fig. S7A–S7C shows the expression pattern of additional genes related to starch and sucrose metabolism. The expression patterns of genes participating in the citrate cycle pathway are identified in Figs. S7D and S7E, which shows that more genes were up-regulated during fruit ripening. More detailed information on these genes is listed in Table S11. The expression of three *CS* (c74887_g1, c78658_g1, and c78658_g3) and one *ACS* gene (c74238_g1) was up-regulated (Fig. S7D), which indicated that the synthesis of citric acid increased during fruit ripening. The up-regulated succinate dehydrogenase gene (c77175_g1) and down-regulated malate dehydrogenase gene (c70484_g1) illustrated that the accumulation of malic acid increased during fruit ripening.

The research on strawberry fruit texture focuses on the cell wall modifying enzymes. In this paper, two DEGs of *endoglucanase CX-like* (*Fa^a^EG*) (c8256_g1) and *endoglucanase 24-like* (*Fa^b^EG*) (c66070_g2) were selected to verify their expression patterns in strawberry ripening process. The results showed that the expression level of *Fa^a^EG* and *Fa^b^EG* was higher in the TF and WT (Figs. 5G and 5H), which was not inconsistent with the expression pattern in transcriptome data (Figs. S6G and S6H). Therefore, these two genes cannot be used to study the softening of strawberry fruit.

### Genes involved in ubiquitin mediated proteolysis associated with the fruit development and ripening process

**Figure 9:**
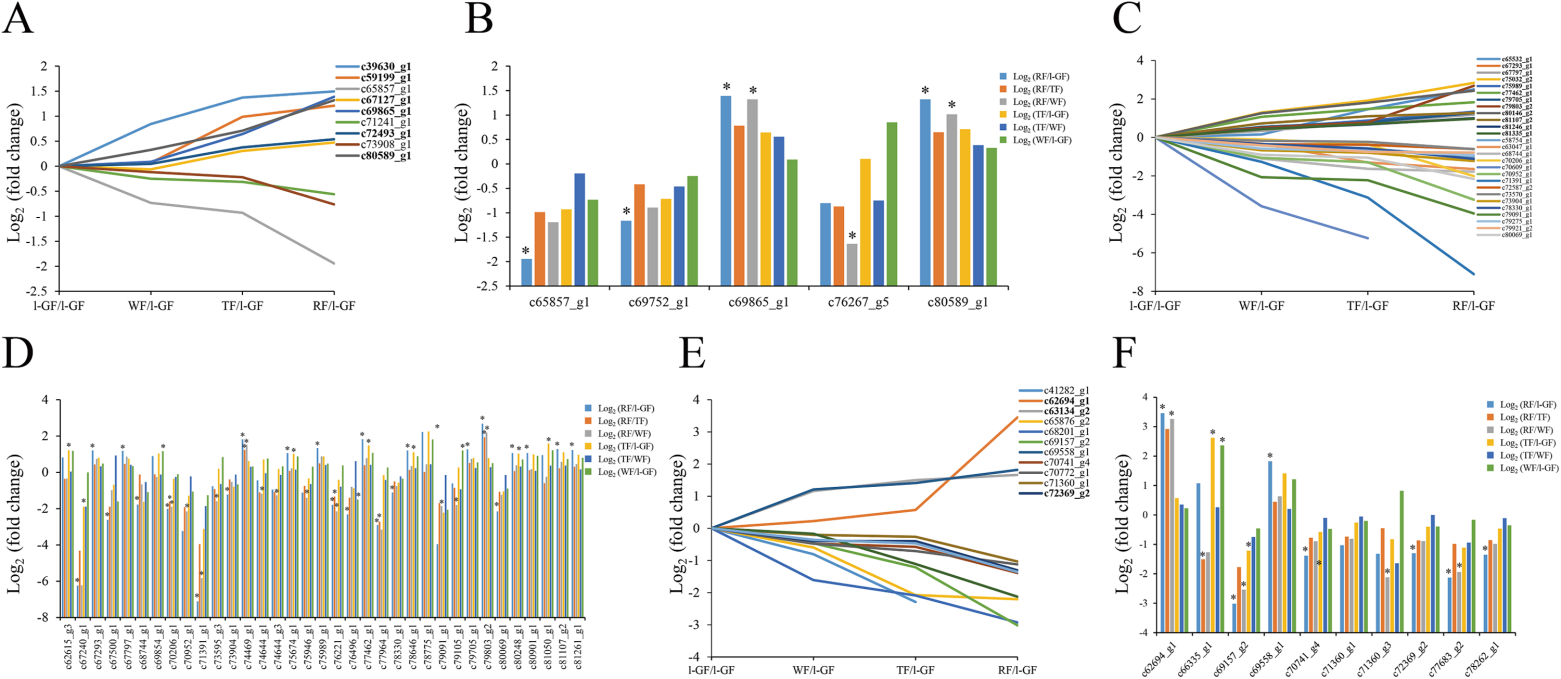
The expression pattern of genes involved in the ubiquitin mediated proteolysis pathway and MADS-box transcript factors. (A) The relative expression of up- and down-regulated genes of ubiquitin conjugating enzyme. (B) The expression pattern of DEGs of ubiquitin conjugating enzyme. (C) The relative expression of up-regulated and down-regulated genes of ubiquitin protein ligase. (D) The expression pattern of DEGs of ubiquitin protein ligase. (E) The relative expression of up- and down-regulated genes of MADS-box transcript factors. (F) The expression pattern of DEGs of MADS-box transcript factors. Black fonts indicate the up-regulated gene ID. The asterisk (*) indicates that the gene is satisfied the differentially expression analysis criteria (padj < 0.05 and log_2_ (fold change) ≥ 1 or log_2_ (fold change) ≤ −1) in the corresponding comparative combination.

Ubiquitin-activating enzyme (E1), ubiquitin-conjugating enzyme (E2), and ubiquitin-protein ligase (E3) are the three major enzymes in ubiquitin mediated proteolysis. The specificity of target proteins is determined by E2 and E3 in ubiquitin mediated proteolysis (Schwechheimer & Calderon Villalobos, 2004; Stone & Callis, 2007; Wang et al., 2014). Only nine E1 proteins were identified in this transcriptome data, and one E1 DEGs (c69468_g2) was only up-regulated in RF/l-GF. Some E2 and E3 proteins were analyzed based on their expression pattern in the transcriptome data (Figs. 9A–9D; Tables S11). Six and three E2 genes were up- and down-regulated, respectively, during strawberry ripening (Fig. 9A). Among the DEGs annotated as E2, the expression of two DEGs (c65857_g1 and c69752_g1) was down-regulated in RF/l-GF and that of one DEG (c76267_g5) was down-regulated in RF/WF. The expression of two E2 DEGs (c69865_g1 and c80589_g1) were all up-regulated in RF/l-GF and RF/WF (Fig. 9B). The expression of 12 E3 genes was up-regulated (Fig. 9C; Table S11), and that of 15 genes decreased during fruit ripening. The differential expression analysis results for E3 showed that the expression of three DEGs (c67240_g1, c80832_g1, and c68571_g1) decreased and that of one DEG (c37206_g1) increased in TF/l-GF. In the RF/l-GF combination, the expression of six DEGs (c67240_g1, c77964_g1, c68571_g1, c77964_g1, c70427_g1, and c79627_g3) decreased and that of three DEGs (c73766_g1, c80901_g1, and c81107_g2) increased during fruit ripening. The expression of two E3 DEGs (c63405_g1 and c68571_g1) decreased in the TF/WF combination. The expression of an E3 DEG (c73766_g1) increased in both RF/WF and RF/TF and that of two DEGs (c70427_g1 and c77964_g1) decreased in RF/WF and RF/TF, respectively (Fig. 9D; Table S11). Based on the above results, the expression quantity of E2 DEGs in the later stage (RF) was significantly different from that of the early stages (l-GF and WF), and no E2 DEG was identified in any other combination. The down-regulated and up-regulated DEGs of E2 and E3 were possibly closely related to the fruit ripening process.

The expression patterns of MADS-box transcription factors were studied (Figs. 9E and 9F; Tables S11). The transcriptional level of most MADS-box transcription factors was down-regulated during fruit ripening. The expression pattern analysis showed that three and eight MADS-box transcription factors increased and decreased during fruit ripening, respectively (Fig. 9E). Among DEGs of MADS-box transcription factors in each combination, the expression of five DEGs (c70741_g4, c72369_g2, c71360_g1, c69175_g1, and c77683_g2) was down-regulated and that of three DEGs was up-regulated in the RF/l-GF comparison (Fig. 9F; Table S11). In the RF/WF comparison, the expression of three DEGs (c77683_g2, c71360_g3, and c69157_g2) was down-regulated and that of two DEGs (c70335_g1 and c62694_g1) was up-regulated during fruit ripening. In the TF/l-GF comparison, the expression of one DEG (c69157_g2) was down-regulated and that of two DEGs (c70335_g1 and c66335_g1) was up-regulated (Table S11). The expression of two MADS-box DEGs (c62694 and c66335) was up-regulated in the RF/TF and WF/l-GF comparisons (Fig. 9F; Table S11). In terms of the above results, more DEGs were found in RF/l-GF and RF/WF than in other comparisons, thus the MADS-box transcription factor DEGs were related to fruit ripening to some extent. According to the known functions of MADS-box transcription factors in fruit ripening, further study of MADS-box transcription factors might lead to a new discovery pertinent to the regulation of fruit ripening.

The expression patterns of some *E2*, *E3*, and *MADS-box* genes were analyzed by qRT-PCR. The results showed that the expression levels of *E2* and ^*a*^E3 were the highest at the TF stage of strawberry and that of ^*b*^E3 was up-regulated during fruit ripening (Figs. 5I–5N). The expression patterns of *Fa^a^EG*, *Fa^a^E2*, *Fa^b^E2*, and *Fa^b^E3* were similar, suggesting that they might have the same function in the fruit ripening process. The expression of *FaMADS-box* decreased significantly during fruit ripening, as shown in Fig. 5O. Combining gene expression patterns in the transcriptome data (Figs. S6I–S6O), More work is required to discover and verify the regulatory mechanisms and functions of E2, E3, and MADS-box transcription factors in the fruit development and ripening process.

## Discussion

In previous studies, RNA-Seq technology has been used to study fruit development and ripening (Kang et al., 2013; Pillet et al., 2015; Li et al., 2016; Estrada-Johnson et al., 2017; Hartl et al., 2017). In this study, 91,790 unigenes were obtained. In addition, 6,608 DEGs were identified to analyze the changes in fruit characteristics with strawberry development and ripening. When our transcriptome data were compared with the transcriptome assembly results of octoploid strawberry in a previous study (Sanchez-Sevilla et al., 2017), fewer reads were used in mapping in this data set (Table 1) due to the sequencing technology at that time and the experimental design, but more genes with FPKM > 0.3 were identified than in the previous study (Sanchez-Sevilla et al., 2017) (Table S3). The unigenes with FPKM > 0.3 were considered to be expressed (Mortazavi et al., 2008; Trapnell et al., 2010; Ho et al., 2012), and the subsequent differentially expression analysis was based on the expressed unigenes.

The most intuitive indicator of strawberry ripening is the coloring. The synthesis mechanism of anthocyanins derived from the plant secondary metabolite pathway of flavonoid biosynthesis, has been extensively studied in strawberry (Manning, 1998; Castellarin & Di Gaspero, 2007; Niu et al., 2010). The high expression of genes such as *CHS*, *CHI*, *F3H*, and *DFR* increases the accumulation of anthocyanin content during fruit ripening (Almeida et al., 2007; Salvatierra et al., 2010; Jiang et al., 2012; Zhang et al., 2015; Hartl et al., 2017). Except for the down-regulated *F3′M* (c72378_g2), which accelerated the accumulation of pelargonidin, the other up-regulated DEGs in anthocyanin biosynthesis promoted fruit coloring and ripening (Fig. 4). The MYB-bHLH-WD40 transcription factors complex regulates the biosynthesis of anthocyanins (Schwinn et al., 2006; Allan, Hellens & Laing, 2008; Hichri et al., 2011; Schaart et al., 2013). *FaMYB10* plays a positive regulatory role in the flavonoid/phenylpropanoid pathway (Lin-Wang et al., 2014; Medina-Puche et al., 2014). *FaMYB1* is described as a transcriptional repressor and represses the biosynthesis of anthocyanins in strawberry (Aharoni et al., 2001). Among the transcription factors of bHLH and WD40, FabHLH33, FabHLH3 and FaTTG1 transcription factors interact with the MYB transcription factors to play important roles in proanthocyanidin and anthocyanin biosynthesis (Schaart et al., 2013). In this study, the up-regulated expression of R2R3 MYB transcription factor (MYB10) (c76851_g2) was positively correlated with its function in anthocyanin biosynthesis. Consistent with a negative regulatory function in anthocyanin biosynthesis (Aharoni et al., 2001), FaMYB1R1 (c75011_g1) was down-regulated. The expression of bHLH33 (c75633_g2) and bHLH3-like (c78773_g1) was down-regulated during fruit ripening. No difference was detected in expression of WD40 during the four fruit ripening stages. The function of those genes related to anthocyanin biosynthesis requires future verification. Strawberry fruits release a special fragrance in the ripening process. AAT was shown to participate in the synthesis of strawberry fruit aroma, and its expression was observed to be up-regulated/increased during fruit ripening process (Perez et al., 1996; Cumplido-Laso et al., 2012). The expression pattern of AAT was significantly up-regulated during fruit ripening, based on qRT-PCR, which was consistent with the expression pattern in this transcriptome data set and that of the previous study.

Little is known of the functional mechanism of ubiquitin mediated proteolysis in strawberry fruit ripening. In a previous study on banana, *MuUBA*, the ubiquitin-activating enzyme E1 gene, and *MuMADS1* showed high expression in the four ovule stage, and the expression levels were stimulated by exogenous ETH and suppressed by 1-methylcyclopropene in banana (Liu et al., 2013). These results indicated that the interaction of MuMADS1 and MuUBA might play an important role in post-harvest banana fruit ripening. SIUBC32 encodes an E2 ubiquitin-conjugating enzyme and five E2s as direct targets of ripening-inhibitor (RIN) were identified, which uncovered a novel regulatory function of proteins in ubiquitin mediated proteolysis in tomato fruit ripening (Wang et al., 2014). Based on the above findings, 34 putative *CpUBC* genes are identified in the papaya genome (Jue et al., 2017). The expression patterns of these genes showed the expression level of 13 *CpUBC* genes increased at one ripening stage and that of two *CpUBC* genes decreased at two ripening stages, which indicated the possible regulatory function of E2s in papaya fruit ripening. Additionally, ubiquitin mediated proteolysis participates in fruit ripening found based on microRNA analysis (Bi et al., 2015; Zeng et al., 2015). In this study, the analysis of E2 DEGs in different comparative combinations of fruit ripening stages showed that the expression levels of E2 28-like and E2 4-like decreased from l-GF to RF. The expression of E2 5-like and E2 23-like increased from l-GF to RF (Table S11). The expression of E3 DEGs of S-phase kinase-associated protein 1 and ubiquitin-protein ligase TRIP12 increased and that of the other E3 DEGs decreased during fruit ripening (Table S11). The expression patterns of E2 and E3 DEGs in qRT-PCR were not consistent with those in the transcriptome data. Based on the differentially expressed patterns of these genes in the transcriptome data, their functional mechanisms in regulating fruit ripening require in-depth research.

The texture of strawberry fruit changes significantly changes during fruit ripening. The regulatory factors that regulate the synthesis of enzymes related to fruit softening, play important roles in fruit ripening (Youssef et al., 2012). MADS-box transcription factors are key elements of the genetic networks that control flower and fruit development, and currently, a pivotal regulatory effect of these transcription factors on fruit ripening is widely reported. Recently, *MdMADS1* was found to inhibit fruit coloration and regulate apple fruit ripening (Ireland et al., 2013; Feng et al., 2016). *TAGL1*, a MADS-box transcription factor gene, controls several aspects of tomato fruit ripening by regulating carotenoid synthesis, ETH signaling pathway, cell cycle regulation, flavonoid and lignin biosynthesis, and cuticle development (Garceau, Batson & Pan, 2017). The suppressed expression of *SlMBP8*, a MADS-box gene, promotes carotenoid and ETH biosynthesis and induces the expressions of cell wall metabolism genes, which ultimately accelerate tomato fruit ripening (Yin et al., 2017). The MADS-box genes of *MaMADS24* and *MaMADS49* regulate the fruit development and ripening process by interacting with MaMADS proteins themselves and ETH signal transduction, biosynthesis-related proteins, starch biosynthesis proteins, and metabolism-related proteins (Liu et al., 2013; Hu et al., 2017). The *PrupeSEP1* gene, a subfamily of MADS-box transcription factors, regulates fruit ripening and softening by exhibiting similar expression patterns of cell wall modification-related genes and N-glycan processing genes in melting flesh peach (Li et al., 2017a). Transcriptome profiles analysis revealed that the silence of fruit-related gene *SEP1/2-like* (*FaMADS9*) leads to the inhibition of normal development and ripening in strawberry achenes (Seymour et al., 2011; Qin et al., 2012; Wang et al., 2014). In our study, the differential expression of MADS-box proteins SVP-like, ZMM17-like, CMB1-like, and MADS-box 17-like, among others which has not been reported in other studies, was identified in the strawberry fruit development and ripening process (Table S11).

RIN, a MADS-box transcription factor, is a key regulator of the ripening gene expression network and has hundreds of target genes that can regulate changes in fruit color, flavor, texture, and taste with tomato fruit ripening (Li et al., 2017b). RIN directly binds to the promoters of SIUBC32, who encodes an E2 ubiquitin-conjugating enzyme involved in the regulation of fruit ripening, and a genome-wide survey of the E2 gene family in tomatoes identified five more E2s as direct targets of RIN (Wang et al., 2014). Based on the relevance and possible regulatory role of E2 and MADS-box DEGs in the strawberry fruit ripening process, further work must be performed to verify the function and relationship between ubiquitin mediated proteolysis and MADS-box transcription factors in the fruit ripening process.

## Conclusion

A transcriptome analysis identified many DEGs associated with fruit ripening characteristics. These DEGs were involved in multiple metabolic pathways of flavonoid biosynthesis, ester biosynthesis, starch and sucrose biosynthesis, the citrate cycle, MADS-box transcription factors, and the ubiquitin mediated proteolysis pathway, among others, in the fruit ripening process. The functional analysis and expression patterns of DEGs related to fruit development and ripening characteristics lay the foundation for the development of molecular markers in the cultivation of new strawberry varieties. The results of this study will also contribute to strawberry molecular breeding.

## Supplemental Information

10.7717/peerj.4976/supp-1 Supplemental Information 1 Table S1. URLs, annotation methods and parameters of seven databases. Each data indicates the characteristics, URLs and usage parameters of the seven databases in this manuscript.

10.7717/peerj.4976/supp-2 Supplemental Information 2 Table S2. The information of software version and parameter. The detail information of software that used in the production of all the transcriptome data.

10.7717/peerj.4976/supp-3 Supplemental Information 3 Table S3. The distribution of FPKM values of each library. FPKM: fragments per kilobase of exon per million fragments mapped. FPKM is the most commonly used method of estimating gene expression level, which eliminates the expression level of technical deviation. Those genes whose FPKM > 0.3 were considered to be expressed. The underlined number indicates that the interval contains the value.

10.7717/peerj.4976/supp-4 Supplemental Information 4 Table S4. Primers used in this study. Primer sequence information of candidate genes in quantitative real-time polymerase chain reaction.

10.7717/peerj.4976/supp-5 Supplemental Information 5 Table S5. The annotation results of unigenes in seven databases. Each data indicates the number and percentage of unigenes in corresponding database.

10.7717/peerj.4976/supp-6 Supplemental Information 6 Table S6. The GO classification of unigenes. Each data indicates the classification system information of unigenes and their products, and the number of unigenes in a GO classification trem.

10.7717/peerj.4976/supp-7 Supplemental Information 7 Table S7. The KOG classification of unigenes. Each data indicates the number of unigenes in 26 gene function classes of KOG database.

10.7717/peerj.4976/supp-8 Supplemental Information 8 Table S8. The KEGG classification of unigenes. Each data indicates the number of unigenes that involved in corresponding metabolic pathway of KEGG database.

10.7717/peerj.4976/supp-9 Supplemental Information 9 Table S9. Differential analysis results of genes in different combinations. Each data is used to determine the differentially expressed genes (DEGs). The DEGs with padj < 0.05 and log_2_ (fold change) ≥ 1 are up-regulated, and those with padj < 0.05 and log_2_ (fold change) ≤ −1 are down-regulated. The other genes that do not meet the conditions of padj < 0.05, log_2_ (fold change) ≥ 1 and log_2_ (fold change) ≤ −1 are not DEGs.

10.7717/peerj.4976/supp-10 Supplemental Information 10 Table S10. Detailed information of genes in the flavonoid biosynthesis pathway. Each data indicates the expression data (read_count) of genes in each library that used for cluster analysis in the flavonoid biosynthesis pathway.

10.7717/peerj.4976/supp-11 Supplemental Information 11 Table S11. Detailed information of genes in Results section. Detail information of all the genes in Results section, including the corrected read_count value, differential analysis results and annotation information in each library and combination.

10.7717/peerj.4976/supp-12 Supplemental Information 12 Fig. S1. FPKM interval of all samples. FPKM: fragments per kilobase of exon per million fragments mapped. The percentage of each sample’s corresponding FPKM interval can be used to measure the difference in expression between samples.

10.7717/peerj.4976/supp-13 Supplemental Information 13 Fig. S2. Length distribution of transcripts and unigenes. The *x-axis* represents the length interval of transcript/unigene, and the *y-axis* represents the number of times for each length of the transcript/unigene.

10.7717/peerj.4976/supp-14 Supplemental Information 14 Fig. S3. Characteristics of homology search of Illumina sequences against the Nr database. (A) Percentage of the total homologous sequences of 5 top species against the Nr database; (B) E-value distribution of the top BLASTx hits against the Nr database; (C) Similarity distribution of the top BLASTx hits for each sequence.

10.7717/peerj.4976/supp-15 Supplemental Information 15 Fig. S4. Expression pattern of genes in the flavonoid biosynthetic pathway. (A) Cluster analysis of genes in flavonoid biosynthetic pathway. Expression level was showed by different colors, the redder the higher expression and the bluer the lower. The values of red to blue is Z score. *Z* = (*x*−μ)/σ, in which *x* is the raw data that needs to be standardized, μ is the average value, and σ is the standard deviation. (B) The relative expression of up- and down-regulated genes in flavonoid biosynthetic pathway. Black fonts indicate the up-regulated gene ID. (C) The expression pattern of DEGs in flavonoid biosynthetic pathway. The asterisk (*) indicates that the gene is satisfied the differentially expression analysis criteria (padj < 0.05 and log_2_ (fold change) ≥ 1 or log_2_ (fold change) ≤ −1) in the corresponding comparative combination.

10.7717/peerj.4976/supp-16 Supplemental Information 16 Fig. S5. Expression pattern of MYB and bHLH transcription factors. (A/C) The relative expression of up- and down-regulated MYB and bHLH transcription factors. Black fonts indicate the up-regulated gene ID. (B/D) The expression pattern of DEGs of MYB and bHLH transcription factors. The asterisk (*) indicates that the gene is satisfied the differentially expression analysis criteria (padj < 0.05 and log_2_ (fold change) ≥ 1 or log_2_ (fold change) ≤ −1) in the corresponding comparative combination.

10.7717/peerj.4976/supp-17 Supplemental Information 17 Fig. S6. The expression level of candidate genes in transcriptome data. Each data indicates the expression pattern of candidate genes with strawberry ripening in transcriptome data.

10.7717/peerj.4976/supp-18 Supplemental Information 18 Fig. S7. Expression pattern of genes in starch and sucrose biosynthesis and citrate cycle. (A/B) The relative expression of up- and down-regulated genes in starch and sucrose biosynthesis. (C) The expression pattern of DEGs in starch and sucrose biosynthesis. (D) The relative expression of up- and down-regulated genes in citrate cycle. Black fonts indicate the up-regulated gene ID. (E) The expression pattern of DEGs in citrate cycle. The asterisk (*) indicates that the gene is satisfied the differentially expression analysis criteria (padj < 0.05 and log_2_ (fold change) ≥ 1 or log_2_ (fold change) ≤ −1) in the corresponding comparative combination.

10.7717/peerj.4976/supp-19 Supplemental Information 19 Assembled transcriptome data of unigenes. The information of unigene id and base sequence of unigenes.

## Additional Information and Declarations

### Data Availability

The following information was supplied regarding data availability: The Illumina reads have been deposited in the Sequence Read Archive (SRA) database at NCBI (http://www.ncbi.nlm.nih.gov/sra) and are available under study accession number SRP111905.
