# Supplementary figures and images for: Transcriptome profiling by RNA-Seq reveals differentially expressed genes related to fruit development and ripening characteristics in strawberries (Fragaria × ananassa)
Source: PeerJ. 2018 Jun 27;6:e4976. doi: 10.7717/peerj.4976 (PMC6026456; doi:10.7717/peerj.4976)

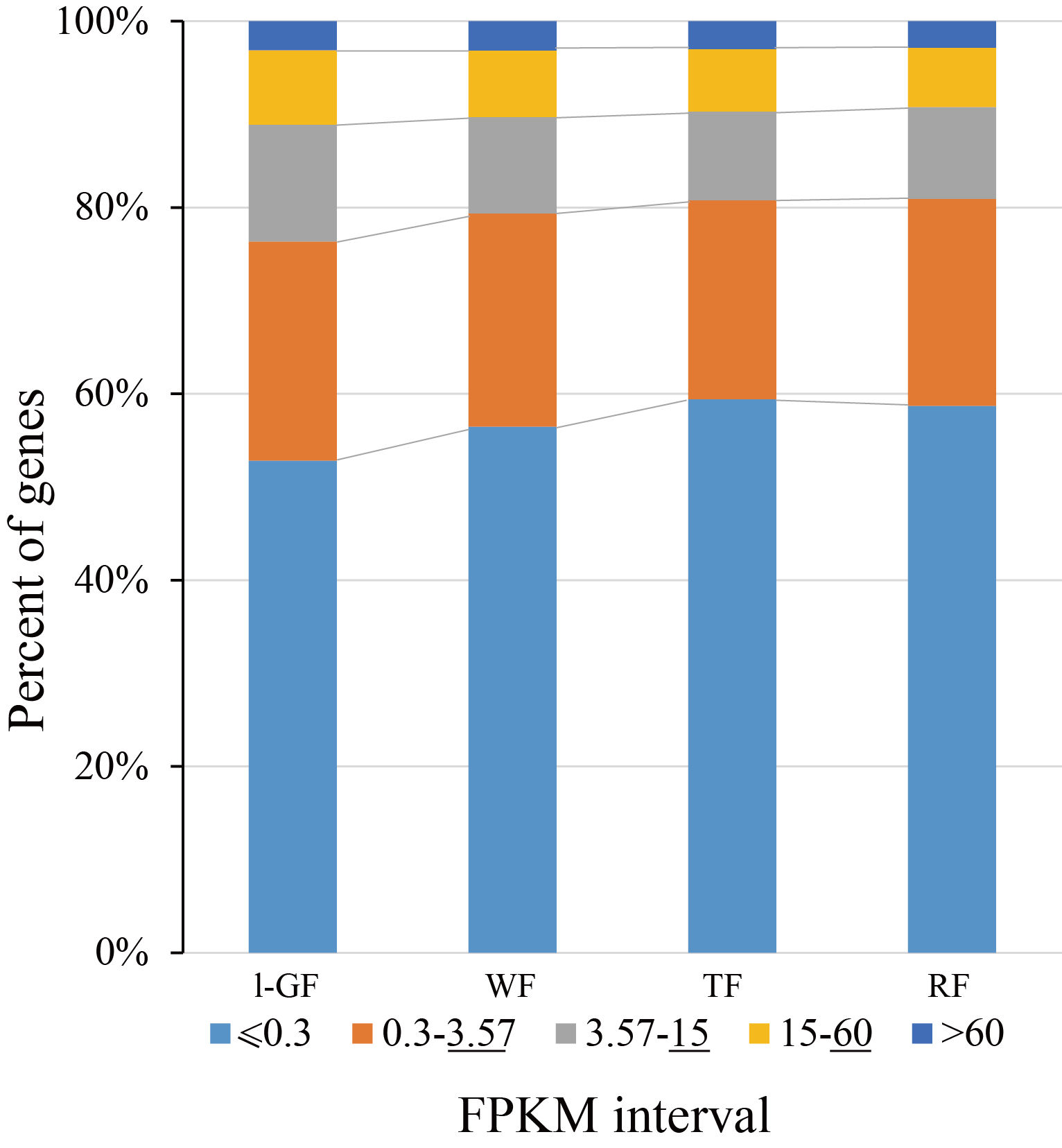

Supplement: Supplemental Information 12 — FPKM: fragments per kilobase of exon per million fragments mapped. The percentage of each sample’s corresponding FPKM interval can be used to measure the difference in expression between samples. [file peerj-06-4976-s012.png]

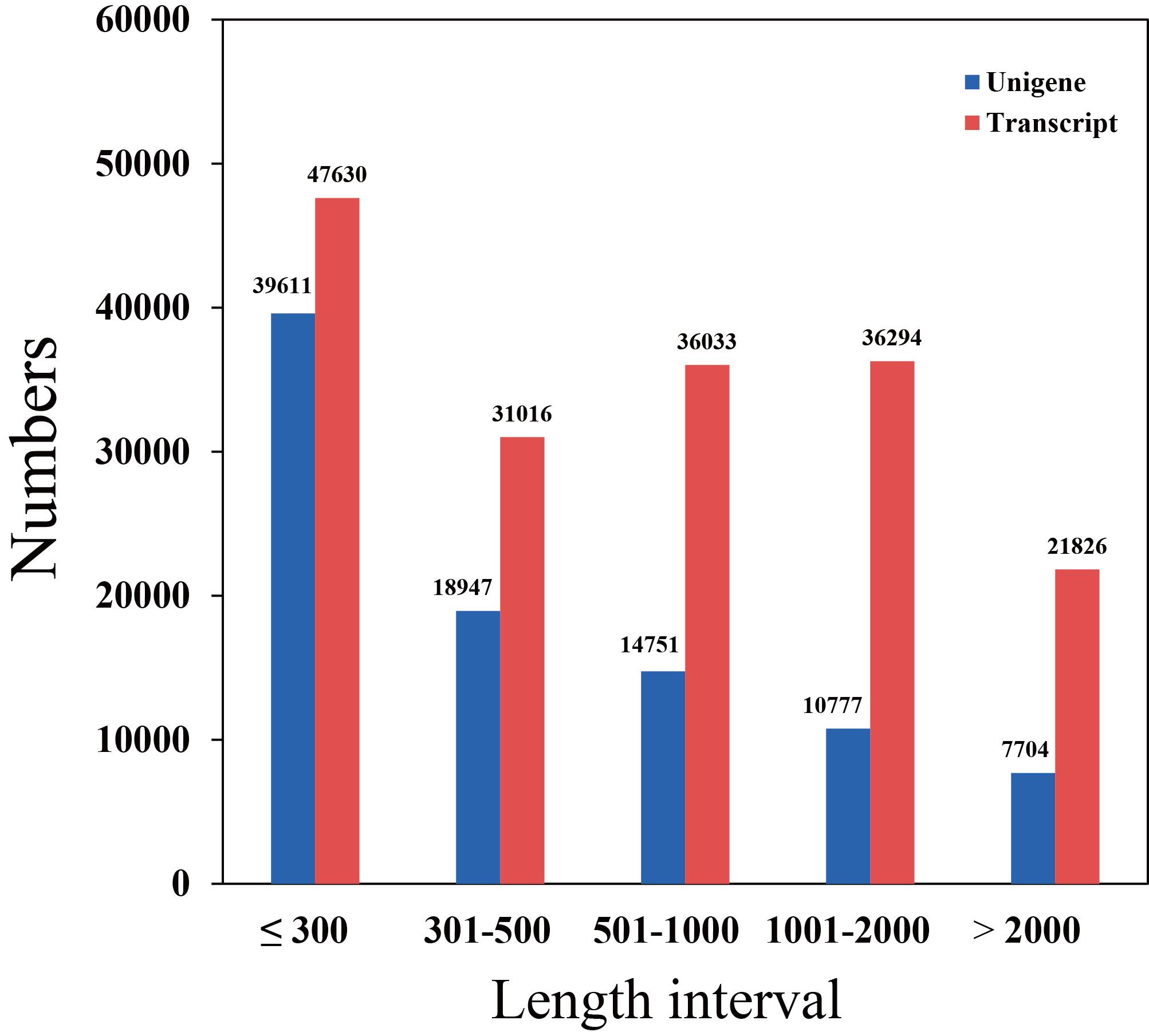

Supplement: Supplemental Information 13 — The x-axis represents the length interval of transcript/unigene, and the y-axis represents the number of times for each length of the transcript/unigene. [file peerj-06-4976-s013.png]

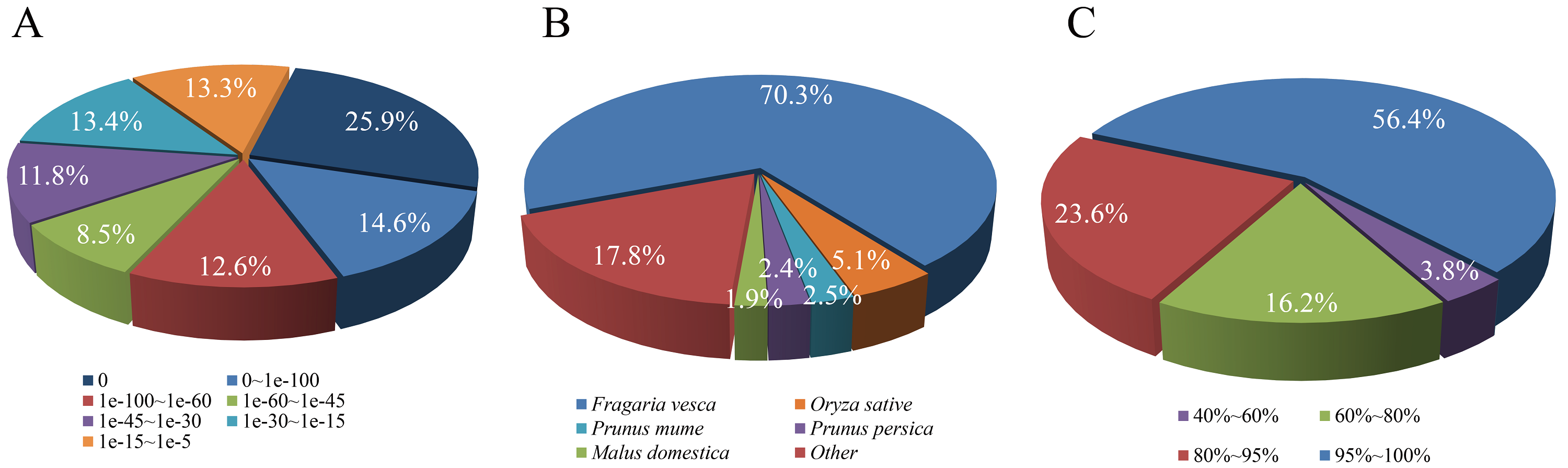

Supplement: Supplemental Information 14 — (A) Percentage of the total homologous sequences of 5 top species against the Nr database; (B) E-value distribution of the top BLASTx hits against the Nr database; (C) Similarity distribution of the top BLASTx hits for each sequence. [file peerj-06-4976-s014.png]

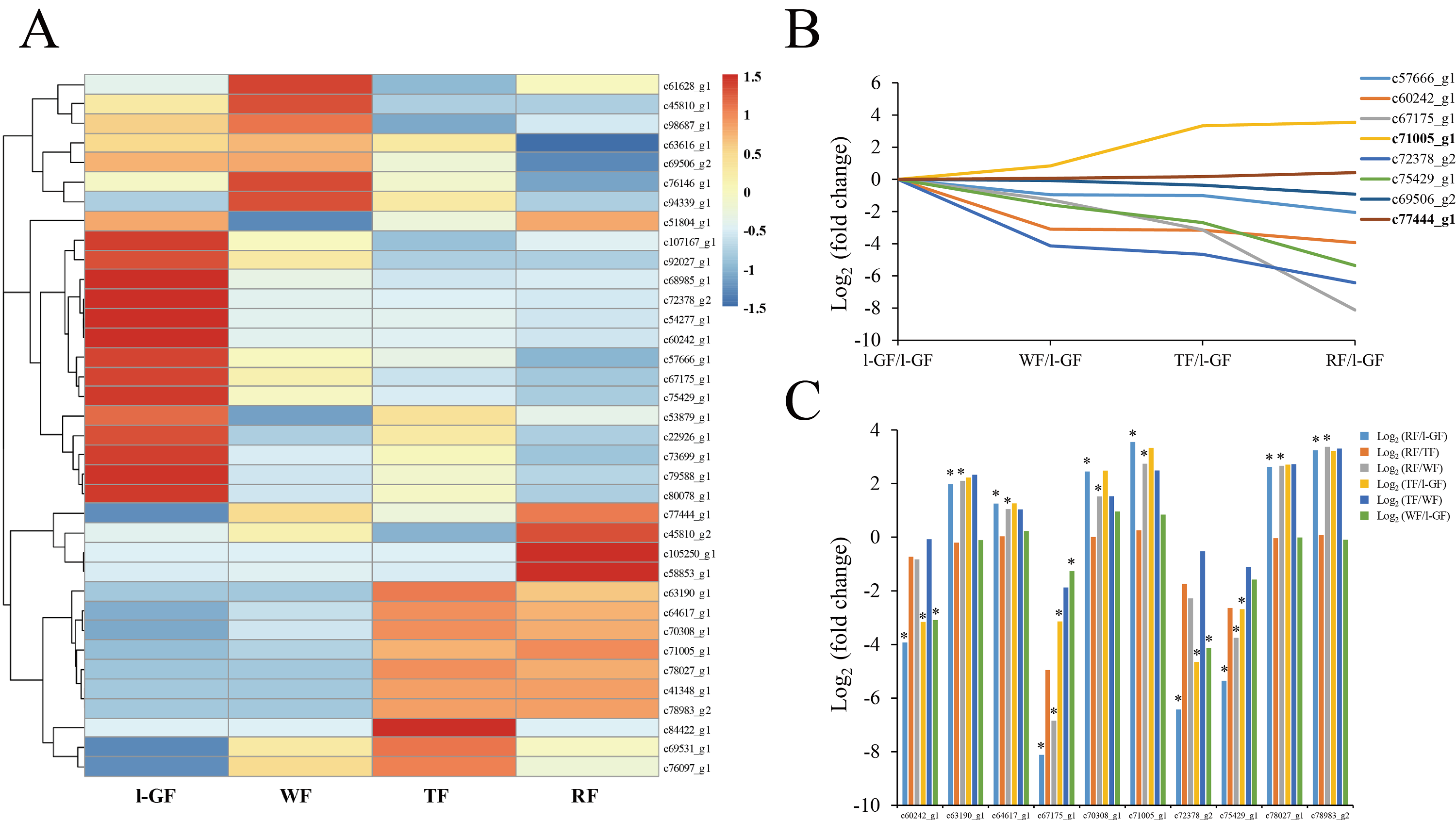

Supplement: Supplemental Information 15 — (A) Cluster analysis of genes in flavonoid biosynthetic pathway. Expression level was showed by different colors, the redder the higher expression and the bluer the lower. The values of red to blue is Z score. Z = (x−μ)/σ, in which x is the raw data that needs to be standardized, μ is the average value, and σ is the standard deviation. (B) The relative expression of up- and down-regulated genes in flavonoid biosynthetic pathway. Black fonts indicate the up-regulated gene ID. (C) The expression pattern of DEGs in flavonoid biosynthetic pathway. The asterisk (*) indicates that the gene is satisfied the differentially expression analysis criteria (padj < 0.05 and log2 (fold change) ≥ 1 or log2 (fold change) ≤ −1) in the corresponding comparative combination. [file peerj-06-4976-s015.png]

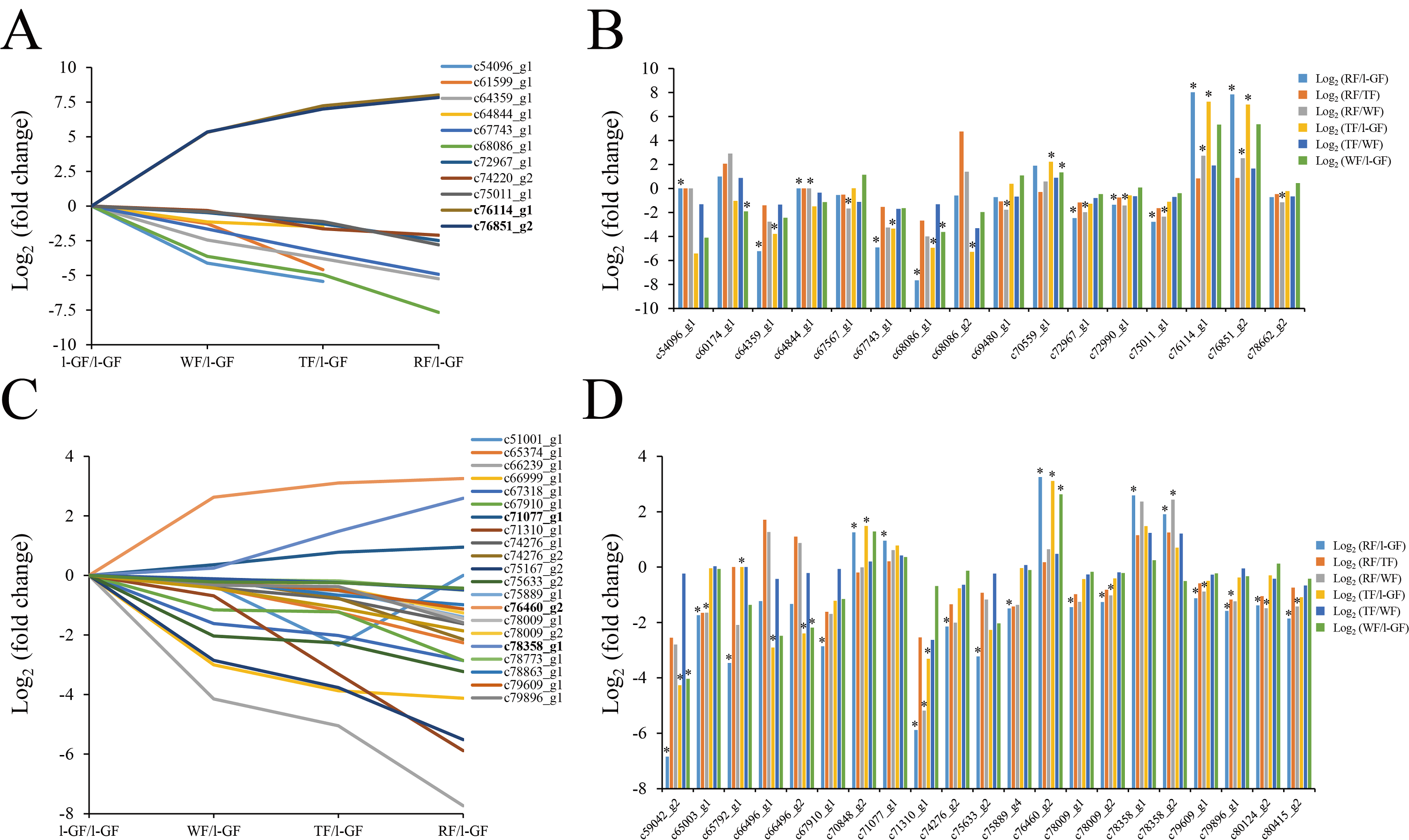

Supplement: Supplemental Information 16 — (A/C) The relative expression of up- and down-regulated MYB and bHLH transcription factors. Black fonts indicate the up-regulated gene ID. (B/D) The expression pattern of DEGs of MYB and bHLH transcription factors. The asterisk (*) indicates that the gene is satisfied the differentially expression analysis criteria (padj < 0.05 and log2 (fold change) ≥ 1 or log2 (fold change) ≤ −1) in the corresponding comparative combination. [file peerj-06-4976-s016.png]

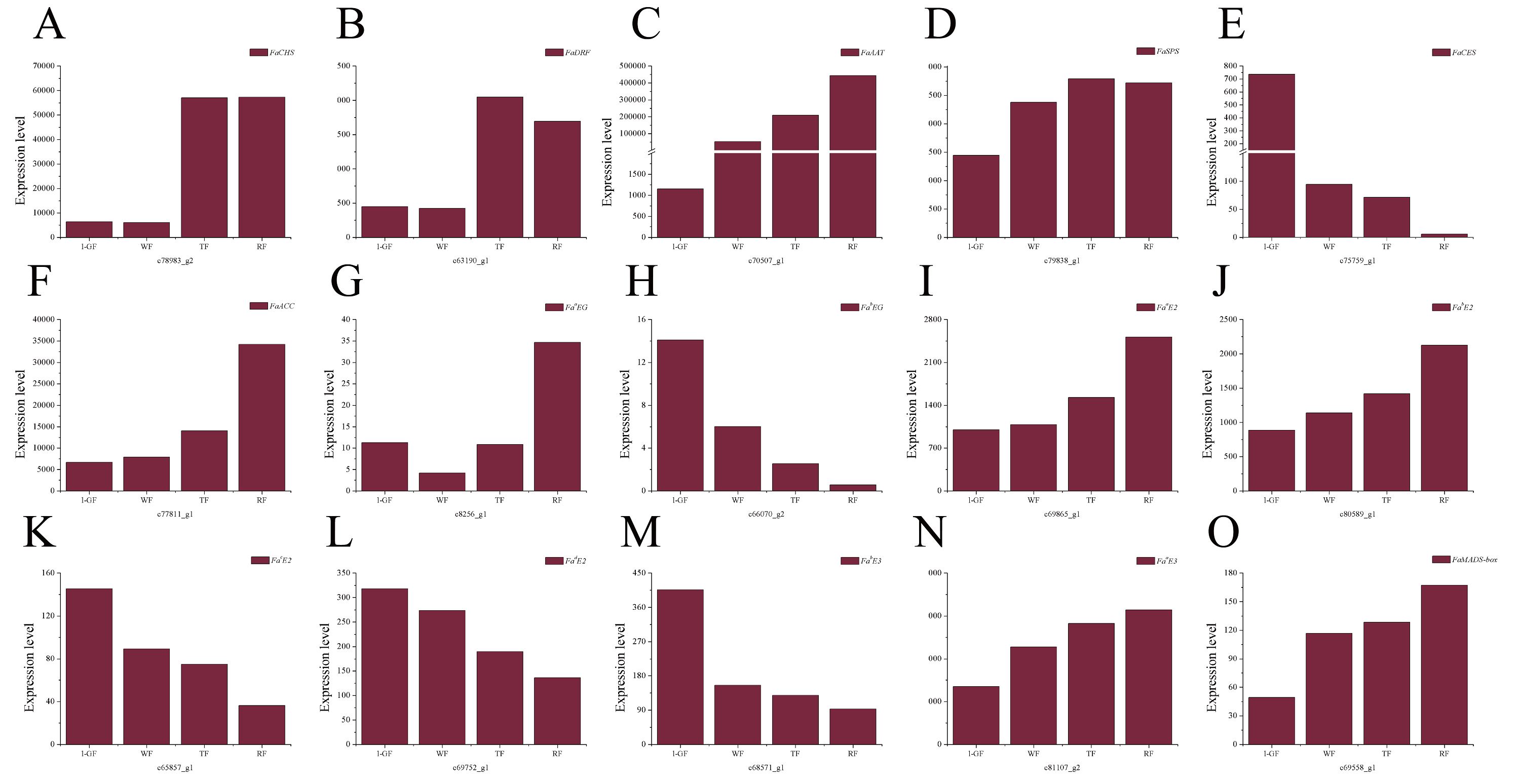

Supplement: Supplemental Information 17 — Each data indicates the expression pattern of candidate genes with strawberry ripening in transcriptome data. [file peerj-06-4976-s017.png]

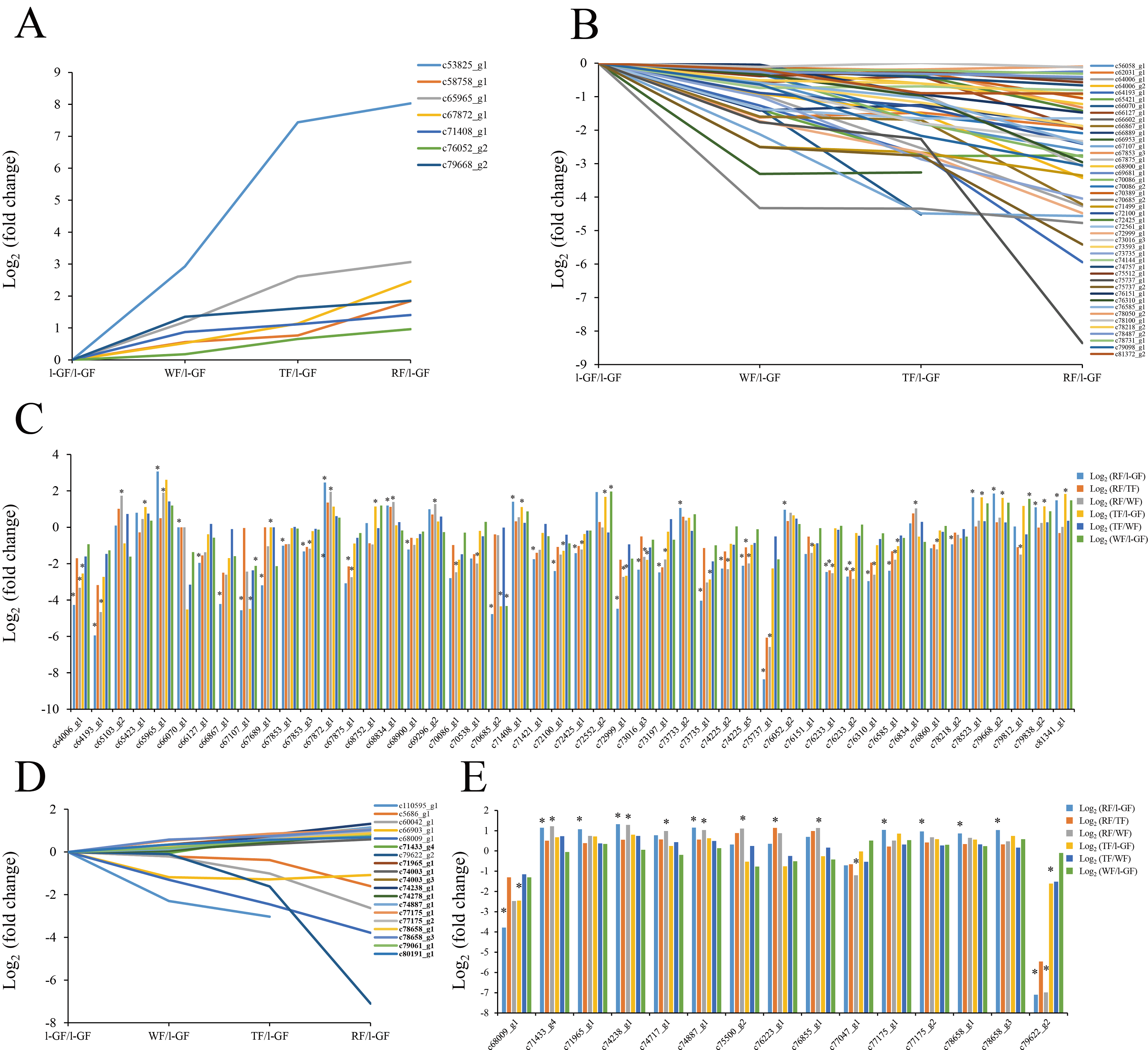

Supplement: Supplemental Information 18 — (A/B) The relative expression of up- and down-regulated genes in starch and sucrose biosynthesis. (C) The expression pattern of DEGs in starch and sucrose biosynthesis. (D) The relative expression of up- and down-regulated genes in citrate cycle. Black fonts indicate the up-regulated gene ID. (E) The expression pattern of DEGs in citrate cycle. The asterisk (*) indicates that the gene is satisfied the differentially expression analysis criteria (padj < 0.05 and log2 (fold change) ≥ 1 or log2 (fold change) ≤ −1) in the corresponding comparative combination. [file peerj-06-4976-s018.png]
